# Identification of novel small molecule-based strategies of COL7A1 upregulation and readthrough activity for the treatment of recessive dystrophic epidermolysis bullosa

**DOI:** 10.1038/s41598-024-67398-8

**Published:** 2024-08-16

**Authors:** Irene Jover, Maria C. Ramos, María José Escámez, Estrella Lozoya, José R. Tormo, Diana de Prado-Verdún, Ángeles Mencía, Mercè Pont, Carles Puig, Marie-Helene Larraufie, Cristina Gutiérrez-Caballero, Fernando Reyes, Juan Luis Trincado, Vicente García-González, Rosario Cerrato, Miriam Andrés, Maribel Crespo, Francisca Vicente, Nuria Godessart, Olga Genilloud, Fernando Larcher, Arsenio Nueda

**Affiliations:** 1 R&D Centre, Almirall S.A., Laureà Miró 408-410, 08980 Sant Feliu de Llobregat, Barcelona, Spain; 2 Fundación MEDINA, Parque Tecnológico de La Salud, Av. Conocimiento 34, 18016 Granada, Spain; 3 Departamento de Bioingeniería E Ingeniería Aeroespacial (UC3M), División de Biomedicina Epitelial, Centro de Investigaciones Energéticas, Medioambientales y Tecnológicas (CIEMAT), Centro de Investigación Biomédica en Red de Enfermedades Raras, Universidad Carlos III de Madrid (UC3M), Madrid, Spain; 4 Unidad de Innovación Biomédica. Centro de Investigaciones Energéticas, U714-CIBER de Enfermedades Raras (CIBERER-ISCIII), Madrid, Spain; 5 Instituto de Investigación Sanitaria, Fundación Jiménez Díaz (IISFJD), Madrid, Spain; 6 Centro de Investigaciones Energéticas, Medioambientales y Tecnológicas (CIEMAT), Madrid, Spain

**Keywords:** Skin diseases, Drug discovery

## Abstract

Recessive dystrophic epidermolysis bullosa (RDEB) is a rare genetic disease caused by loss of function mutations in the gene coding for collagen VII (C7) due to deficient or absent C7 expression. This disrupts structural and functional skin architecture, leading to blistering, chronic wounds, inflammation, important systemic symptoms affecting the mouth, gastrointestinal tract, cornea, and kidney function, and an increased skin cancer risk. RDEB patients have an extremely poor quality of life and often die at an early age. A frequent class of mutations in RDEB is premature termination codons (PTC), which appear in homozygosity or compound heterozygosity with other mutations. RDEB has no cure and current therapies are mostly palliative. Using patient-derived keratinocytes and a library of 8273 small molecules and 20,160 microbial extracts evaluated in a phenotypic screening interrogating C7 levels, we identified three active chemical series. Two of these series had PTC readthrough activity, and one upregulated C7 mRNA, showing synergistic activity when combined with the reference readthrough molecule gentamicin. These compounds represent novel potential small molecule-based systemic strategies that could complement topical-based treatments for RDEB.

## Introduction

Recessive dystrophic epidermolysis bullosa (RDEB) is a rare genetic disease characterized by skin fragility and scarring with varying prevalence estimations that range between 1.35 and 20.4 cases per million people^1,2^. RDEB is caused by a deficiency in Collagen VII (C7) function resulting from alterations in the COL7A1 gene which can result in nonsense (premature termination codon, PTC), frameshift, splicing, or missense mutations appearing alone or in combinations^3^. Frameshift and splicing mutations can also lead to the appearance of PTC in some cases.

C7 produced by epidermal keratinocytes and dermal fibroblasts, is the main component of anchoring fibrils, which are banded structures that secure stable association of the lower side of the lamina densa to the upper papillary dermis of the skin. The absence of functional C7 is the cause of a profound fragility of the skin and mucous membranes in response to a minor trauma, with constant formation of blisters and erosions^4^. Perpetual cycles of wounding result in persistent inflammation and scarring, and non-healing ulcers often complicated by infections that further prolong the healing process. The pro-inflammatory and pro-fibrotic changes in the absence of C7 lead to an upregulation of TGFβ signaling which drives fibroblast differentiation into myofibroblasts, triggering an increased production of inflammatory cytokines^5^. The recurrent scarring and fibrosis contribute to the development of contractures and pseudosyndactyly that markedly increase disease severity and diminish quality of life. RDEB patients carry a high risk of developing aggressive cutaneous squamous cell carcinoma (SCC), which increases with the severity and chronicity of wound healing^3,6^. In addition to its effect on skin, the absence or deficiency in C7 has a significant systemic impact causing extracutaneous manifestations with affectations in the mouth, gastrointestinal tract, cornea, and kidney, among other organs, which further deteriorates the quality of life of these patients^7^.

It is estimated that premature termination codon (PTC) mutations are present in at least 15–20% of RDEB patients^8^. The more severe GS (generalized severe) form of RDEB, affects ~ 2–10% of patients, most of whom have biallelic PTC mutations resulting in C7 loss of function^2,9,10^. Other less severe forms of RDEB involving PTCs and associated with milder symptoms, often result from combinations of PTCs with other types of mutations^11^. RDEB severity not only depends on the type of COL7A1 mutations and their location but is also influenced by individual factors which can include increased levels of TGFβ or pro-inflammatory IL-6 and MCP-1 cytokines^12^. These factors may also influence the levels of truncated or full-length C7 that are detectable in these patients.

To date, gentamicin is the only small molecule drug that has shown efficacy in RDEB caused by PTC nonsense mutations due to its translational readthrough activity, a long-known property of antibiotics of the aminoglycoside class^13^. A clinical proof of concept study in RDEB patients showed that gentamicin restored C7 levels and improved skin wound closure after topical administration^14,15^, and this has been confirmed in a more recent study in 3 patients using intravenous administration^16,17^. However, gentamicin has not yet been approved for use in patients apparently due to its potential toxicity^13^. Other aminoglycoside and non-aminoglycoside molecules with readthrough activity in PTC-driven genetic diseases have been described and are in various stages of research and development^18^. These include ELX-02, ZKN-0013, SRI-37240, and ataluren, the only marketed readthrough drug approved for the treatment of Duchenne muscular dystrophy caused by PTC mutations^19–25^.

In addition to small molecules, several gene, cell, and protein therapies have been explored as therapeutic approaches for RDEB^8^. One of them is B-VEC topical gene therapy locally delivering COL7A1 cDNA with a non-replicating herpes simplex virus that in clinical studies showed durable wound closure, full-length cutaneous C7 expression, and anchoring fibril assembly with minimal adverse events^26^. This led to its approval in May 2023^27^, representing a breakthrough in the search for curative treatments for RDEB^28^.

Despite these advances, there is still a strong need to address the systemic symptoms affecting RDEB patients. In this context, translational readthrough continues to be a promising strategy to provide a systemic treatment for RDEB caused by PTC mutations which could complement topical treatment options. Translation readthrough strategies take advantage of the higher sensitivity to translational readthrough of misplaced stop codons. This is based on our current knowledge of basal readthrough, as a natural process that is more frequent in PTC mutations (0.01–1%) than in native stop codons (NSCs) (0.001–0.1%) due to the proximity of NSCs to the translation termination complex and surrounding sequences^29^. This mechanistic difference enables achieving selectivity of readthrough-inducing compounds without interfering with NSCs. Another potentially complementary strategy is to inhibit the degradation associated with PTC-containing mRNAs, a process known as nonsense-mediated decay (NMD). Blocking NMD could enhance the effect of basal readthrough^30^ and/or improve the response to therapies aimed at restoring C7 levels in RDEB patients, regardless of the type of mutation affecting them. One example of this approach is amlexanox, a repurposed drug initially indicated for the treatment of aphthous ulcers, which was identified as a potential inhibitor of NMD-mediated COL7A1 degradation^9^. Specifically upregulating COL7A1 mRNA would be a much more selective approach to facilitating readthrough of basal or drug-induced mechanisms for RDEB. A recent study to investigate COL7A1 mRNA upregulators using a combination of CRISPR and small molecule screens identified several genes and pathways related to an increase in C7 mRNA levels, which included toll-like receptor, interferon and calcium uptake signaling pathways and led to the identification of the flavonoid kaempferol as an inducer of C7 expression^31^.

To search for novel chemical and biological starting points compatible with systemic therapies for RDEB that could complement local topical approaches, we have used a phenotypic screening strategy that combines a small molecule and a microbial extract library, as source of bioactive chemical diversity, with patient-derived immortalized skin keratinocytes as a relevant translational model of RDEB. Phenotypic screening is a target-agnostic approach that offers the opportunity to tap into mechanisms of action not only related to the function of individual protein targets but also involving any subcellular complex or macromolecular process that impacts the selected endpoint^32^.

Our goal was to identify molecules capable of restoring the expression of full-length C7 protein in RDEB cells via translational readthrough at PTC mutations, or by COL7A1 mRNA upregulation. Restoration of C7 expression in patient-derived keratinocytes is a direct gain of function surrogate of the main intended outcome of an effective therapy for RDEB, which would be to restore the absence or dysfunction of C7^3^.

Using N-terminus-based detection of total C7 as an endpoint capturing both full-length and truncated C7 protein products, we identified and characterized hits from three chemical series. Representative hits from two of these series showed PTC readthrough activity, inducing partial expression of full-length C7, while the third strongly increased the levels of truncated C7, showing synergistic activity when combined with gentamicin as a representative aminoglycoside with readthrough activity^16,33^.

## Results

### Characterization and validation of C7-deficient RDEB patient-derived and recombinant cell lines

Keratinocytes of consenting patients were isolated from surplus cell-biobank tissue obtained from skin samples harvested for molecular diagnosis after ethical committee approval (see materials & methods). These keratinocytes, carrying different RDEB mutations and genetic backgrounds, were used to generate two immortalized cell lines as described in the materials & methods section. These cell lines were characterized using western blot and an antibody-based AlphaLISA assay recognizing an N-terminal region of C7 (amino acids 36-153) within the non-collagenous 1 domain (NC1), spanning amino acids 17-1253^34^. The western blot analysis allowed us to qualitatively differentiate between truncated and full-length forms of C7 based on their size, while the more sensitive AlphaLISA assay detected both full-length and C-terminally truncated mutant forms of the protein in the patient cell lines. In both cases cells were treated with test compounds and TGFβ2, a known inducer of C7 in human keratinocytes^35^ upregulated in RDEB lesions^36^ which was found to enhance the activity of reference compounds during the development of the AlphaLISA assay (Supplementary information Fig S1).

**Figure 1:**
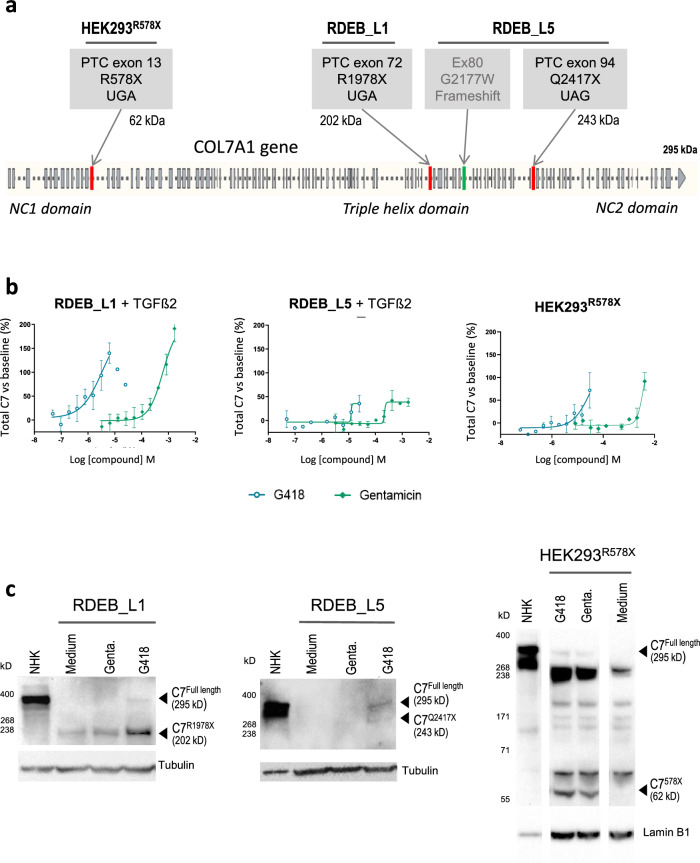
Characterization of recombinant and patient-derived cell lines used in this study. (**a**) Schematic representation of the mutations present in patient-derived RDEB_L1 and RDEB_L5, and recombinant HEK293^R578X^ cells. (**b**) Quantification of total (truncated + full length) C7 in RDEB_L1, RDEB_L5, and recombinant HEK293^R578X^ cells using AlphaLISA (% readthrough: C7 increase vs basal AlphaLISA levels after 48 h in the presence of test compound (G418 or gentamicin) and TGß2 20 ng/mL in the case of RDEB_L1 and L5). Compound concentration is shown as a molar concentration on a logarithmic scale. (**c**) Western blot detection of truncated and full-length C7 in RDEB_L1 and RDEB_L5 patient-derived cells treated with 0.837 mM gentamicin and 36 μM G418, and recombinant HEK293^R578X^ cells treated with 4.18 mM gentamicin and 10 μM G418. NHK, normal human primary keratinocytes. AlphaLISA and western blot are representative results from at least 2 experiments. The image of the western blots in RDEB_L1 and RDEB_L5 was cropped to remove non relevant areas. The image of the HEK293^R578X^ western blot was cropped to remove 3 non-relevant lanes. Full uncropped images are presented in Supplementary information Fig. S2.

Two patient-derived transformed cell lines were generated, RDEB_L1 containing homozygous PTC mutations in exon 72 (PTC, COL7A1:p.R1978X /p.R1978X), and RDEB_L5 containing a PTC in exon 94 in compound heterozygosity with a frameshift mutation in exon 80 resulting from a base insertion (PTC/frameshift, COL7A1: pQ2417X/pG2177Wfs*113) (Fig. 1a). These cell lines were characterized and validated for the phenotypic screen based on their response to aminoglycosides G418 and gentamicin (Fig. 1b, c). Using both cell lines offered 2 different PTC mutation and donor backgrounds adding biological diversity to the screen. In addition to the mutations represented in the 2 selected human patient-derived cells, a mutated version of COL7A1 mRNA (bi-allelic UGA PTC, R578X / R578X), previously shown to respond well to aminoglycosides^37^ was recombinantly generated and stably transfected into HEK293 cells -a cell line that does not express C7- to generate HEK293^R578X^ (Fig. 1a).

In these cell lines, G418 showed superior activity over gentamicin (Tables 1, 2, 3) with an EC_50_ in the AlphaLISA assay (EC_50_ 4.9 µM) that was three orders of magnitude lower than gentamicin (EC_50_ 1.7 mM) in RDEB_L1 (Table 1), and detectable upregulation of full length and truncated C7 in the western blot in RDEB_L1, RDEB_L5, and HEK293^R578X^ cells (Fig. 1b, c). The activity of gentamicin was detectable in the AlphaLISA assay in RDEB_L1 and L5 cells at concentrations > 100 µM (Fig. 1b). In the less sensitive western blot assay, gentamicin showed some upregulation of truncated and full-length forms of C7 only at concentrations > 1 mM (Fig. 1c; supplementary information Fig S7).

**Table 1:** Activity and impact on cellular viability of hit compounds in RDEB_L1 cells.

Compound i.d.	**Increase** **in** **C7** **levels**		**Cytotoxicity**
	EC_50_(rel) [µM]	Emax, % vs baseline at [µM]	% inhibition of viability at [µM]
Gentamicin	1700 ± 480	199 ± 112 at 4100	52.1 ± 11.3 at 4100
G418	4.9 ± 2,4	124 ± 76 at 20	15.5 ± 9.4 at 40
Compound 1	3 ± 1,8	3279 ± 1236 at 12	12 ± 12 at 12
Compound 3	1.9 ± 0.12	2083 ± 29 at 3.1	43 ± 11 at 12
Compound 7	17 ± 6.6	412 ± 80 at 50	10.3 ± 9.9 at 50
Compound 8	40 ± 13	86 ± 13.9 at 100	1.5 ± 4.6 at 100
Chuangxinmycin	n.d.	22 ± 3 at 50	n.d.
3-methyl chuangxinmycin	42	105 ± 13 at 100	6.1 at 100

Total C7 quantified using the AlphaLISA in the presence of 20 ng/mL of TFGß2. Relative EC50 and Emax are shown as arithmetic mean ± standard deviation (SD) values. n.d. not determined. Cytotox. = cytotoxicity measured as % inhibition of viability (ATP levels). Data corresponds to mean ± SD of n = 2–18 experiments except for 3-methyl chuangxinmycin in cytotoxicity (n = 1), where results are the arithmetic average of duplicate determinations (no compound was available for additional determinations).

**Table 2:** Activity and impact on cellular viability of hit compounds in RDEB_L5 cells.

Compound i.d.	Increase in C7 levels		Cytotoxicity
	EC_50_ [µM]	Emax, % vs baseline at [µM]	% inhibition of viability at [µM] or IC_50_
Gentamicin	420 ± 390	41 ± 3.3 at 1600	6.3 ± 2.2 at 8000
G418	12	35.5 at 25	35 ± 0,8 at 80
Compound 1	4.7 ± 0.8	1401 ± 384 at 12.5	n.d.
Compound 3	3.1 ± 0.008	717 ± 229 at 1.25	n.d.
Compound 7	23 ± 11	293 ± 58 at 50	10.7 ± 2.3 at 50

Total C7 quantified using the AlphaLISA in the presence of 20 ng/mL of TFGß2. Relative EC50 and Emax are shown as arithmetic mean ± standard deviation (SD) values. n.d. not determined. Cytotox. = cytotoxicity measured as % inhibition of viability (ATP levels). Data corresponds to mean ± SD of n = 2–7. experiments except for G418 (AlphaLISA) (n = 1), where results are the arithmetic average of duplicate determinations.

In the recombinant HEK293^R578X^ cell line, the activity of both G418 and gentamicin could be detected by AlphaLISA and western blot. In the western blot, the induction of the truncated and full-length C7 forms could be detected together with some additional spurious bands that were probably artifacts of this recombinant system (Fig. 1c).

In patient-derived and recombinant cells, the size of the truncated C7 products detected in the western blot was compatible with their predicted molecular weights, based on the position of the PTC mutations (Fig. 1a, c): 62 kDa (HEK293^R578X^), 202 kDa (RDEB_L1) and 243 kDa (PTC)(RDEB_L5).

### Identification of molecules restoring expression of truncated and full-length C7 in RDEB patient-derived and recombinant C7 deficient cells using phenotypic screening

To identify molecules restoring the expression of C7 in the RDEB patient-derived cell lines RDEB_L1 and RDEB_L5, we applied a phenotypic screening approach using a library that combined a selection of 8,273 representative small molecules from Almirall compound collection and 20,160 microbial extracts from Fundación MEDINA library.

The 8,273 small molecule selection, derived from Almirall compound library was based on compounds that had been filtered for the absence of structural alerts, good physicochemical properties and to maximize structural diversity. This set was the result of merging a selection of 5000 compounds representing more than 30 years of in-house drug discovery projects targeting G-protein coupled receptors (GPCR), ion channels, several families of enzymes including phosphodiesterases and kinases, 200 approved marketed drugs, and 3,435 compounds bearing structural similarity with a selection of reference readthrough and anti-NMD molecules which represented an opportunity to identify additional hits with potential for optimization based on known mechanisms of action (see materials & methods for additional details). A subset of 20,160 crude extracts (10,560 from actinomycetes and 9,600 from fungi) was selected from Fundación MEDINA’s Microbial Natural Products Library to maximize chemical diversity. For actinomycetes, 33 extracts derived from 12 strains which are known producers of aminoglycoside compounds were included to increase the likelihood of known readthrough mechanisms of action also being represented (see materials & methods for additional details).

The small molecule compounds were evaluated at 25 µM, a concentration intended to capture hits with a potency comparable to the relatively potent translational readthrough-active reference aminoglycoside G418 (EC_50_ 4.9–12 µM) and more potent than the reference drug gentamicin (EC_50_ 0.42–1.7 mM) in RDEB_L1 and RDEB_L5 cells (Tables 1, 2). Microbial extracts were evaluated at a relative 20-fold dilution from the original library stock, just below the levels at which an impact on viability started to be detectable for some of the extracts in a preliminary exploratory test. Both the small molecule and microbial extract subsets of the library were evaluated in the presence of 20 ng/mL of TGFβ2.

**Table 3:** Activity and impact on cellular viability of hit compounds in HEK293^R578X^ cells.

Compound i.d.	Increase in C7 levels		Cytotoxicity
	EC_50_ [µM]	Emax, % vs baseline at [µM]	% inhibition of viability at [µM]
Gentamicin	4500 ± 2900	89 ± 17 at 4100	n.d
G418	16 ± 0.09	71 ± 6 at 40	n.d
Compound 1	n.d.	− 7% ± 8 at 50	7.3 ± 14 at 50
Compound 7	60 ± 4.6	96 ± 35 at 100	−1.3 ± 21 at 100
Compound 8	n.d.	10± 13 at 100	4.6 ± 4.5 at 100
3-methyl chuangxinmycin	4.6	58 at 100	−3% at 100

Total C7 quantified using the AlphaLISA. Relative EC50 and Emax are shown as arithmetic mean ± standard deviation (SD) values. n.d. not determined. Cytotox. = cytotoxicity measured as % inhibition of viability (ATP levels). Data corresponds to mean ± SD of n = 2–6 experiments except for 3-methyl chuangxinmycin (n = 1), where results are the arithmetic average of duplicate determinations (no compound was available for additional AlphaLISA determinations).

A gain of signal detection of C7 protein based on the AlphaLISA assay endpoint was used for the phenotypic screening. This had the advantage of minimizing the likelihood of identifying hits with cytotoxic properties, as impaired viability due to cytotoxicity would be expected to have an impact on the cell translational machinery, precluding the expression of C7.

**Figure 2:**
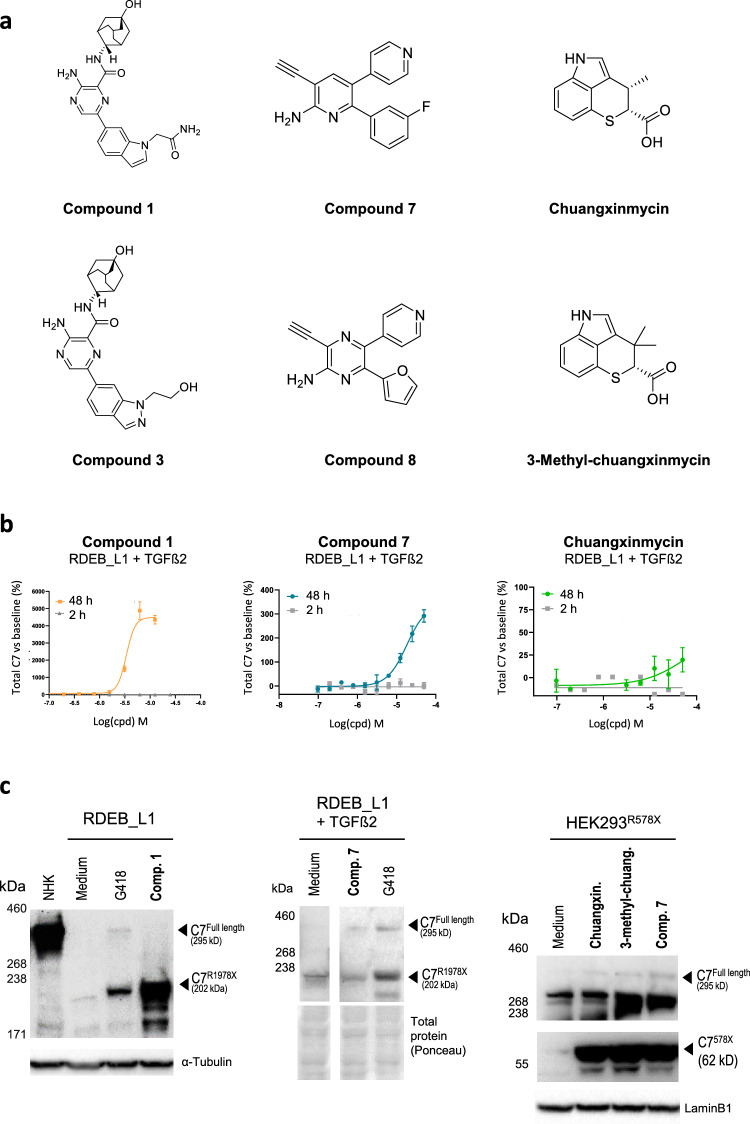
Identification of chemical series upregulating levels of full length and truncated C7. (**a**) Structure of representative molecules from the three chemical series. (**b**) Dose-response confirmation of activity at 2 h and 48 h for the indicated compounds in RDEB_L1 cells in the presence of 20 ng/mL of TFGß2. (**c**) Analysis by western blot of reference and representative compounds from the three chemical series in the indicated cell lines at the following concentrations: 36 μM G418 (left panel), 40 μM G418 (middle panel), 10 μM **compound** **1**, 50 μM **compound** **7,** 50 μM **chuangxinmycin** and 100 μM **3-methyl-chuangxinmycin,** in the presence of 20 ng/mL TGFβ2 in the western blot in the middle panel. AlphaLISA and western blot results are representative from at least 2 experiments except for **chuangxinmycin** western blot which was n=1 due to the limited amount of compound available. Western blots images were cropped for clarity: one overexposed lane was removed in the image of the middle panel. Uncropped images are presented in supplementary information **Fig.** **S3**.

Following the primary screen, the activity of the hits was confirmed in a dose–response study by AlphaLISA, and their potential assay interference was verified by evaluating their activity in the same assay in the absence of cells. Lastly, three chemical series were selected (Fig. 2a), two derived from the small molecule subset of the library, one represented by **compound** **1** and **compound** **3**, and the other by **compound** **7** and **compound** **8**^38^. **Compound** **8** was not in the initial subset included in the library but was identified after a structural similarity search based on **compound** **7**, as part of a hit expansion exercise based on hits from both series, which identified actives in both series. The third chemical series, derived from the microbial extracts subset of the library following extensive fractionation and purification of the active extracts identified as hits, was represented by the antibiotic **chuangxinmycin**^39^ and related compound **3-methyl-chuangxinmycin**^40^. In the AlphaLISA assay hits from the small molecule-derived series were active upregulating total C7 levels in the 3 cell lines evaluated, RDEB_L1 and RDEB_L5 patient-derived, and HEK293^R578X^ recombinant cells (Tables 1, 2, 3). The natural product hits **chuangxinmycin** and **3-methyl-chuangxinmycin** derived from the microbial extracts library were active in RDEB_L1 and HEK293^R578X^ cells. They could not be evaluated in RDEB_L5 cells due to their limited availability.

As an additional assay-interference control, the activity of the most representative compounds from each series, **compound** **1**, **compound** **7,** **chuangxinmycin,** and **3-methyl-chuangxinmycin**, was evaluated in dose–response AlphaLISA assays at 2 h and 48 h in RDEB_L1 cells. This was intended as verification of biological activity, given that a progressive increase in C7 protein or mRNA levels over time would be expected for a biological mechanism-mediated effect, as opposed to a non-specific signal resulting from assay interference, which would be detectable at the initial time point of 2 h (Fig. 2b). In this assay **3-methyl-chuangxinmycin** showed some levels of interference at 2 h at the lower concentrations tested (see supplementary information Fig. S4).

Western blot analysis of the C7 products that were being induced in RDEB_L1 cells by the representative hit compounds, showed that **compound** **1** was only upregulating the truncated form of C7, while **compound** **7**, **chuangxinmycin** and **3-methyl-chuangxinmycin**, evaluated in RDEB_L1 and HEK293^R578X^ cells, partially upregulated full-length C7 (Fig. 2c). These results indicated that **compound** **1** did not have readthrough activity, while **compound** **7**, **chuangxinmycin,** and **3-methyl-chuangxinmycin** were inducing PTC readthrough to yield detectable full-length C7.

The potency, expressed as EC_50_ and Emax in the AlphaLISA assay quantifying total C7 protein, was highest in the **compound** **1** **/** **compound** **3** series which also had a higher impact on viability (using ATP as an endpoint) in RDEB_L1 cells than in normal human primary keratinocytes, as seen with **compound** **3** (Table 1**,** supplementary information Table S1). **Compound** **7** and **chuangxinmycin** / **3-methyl-chuangxinmycin** Emax values in the AlphaLISA assay were more comparable to those of aminoglycosides readthrough reference compounds G418 and gentamicin (Tables 1, 2, 3).

Analysis of the pharmacological signature of **compound** **1** **and** **compound** **7** indicated that at the concentrations relevant for C7 upregulation (low micromolar range and above) (Tables 1, 2, 3) they were expected to fully inhibit their initially annotated pharmacological targets plus some additional off-targets, as revealed for **compound** **7** upon further characterization (Table 4).

**Table 4:**
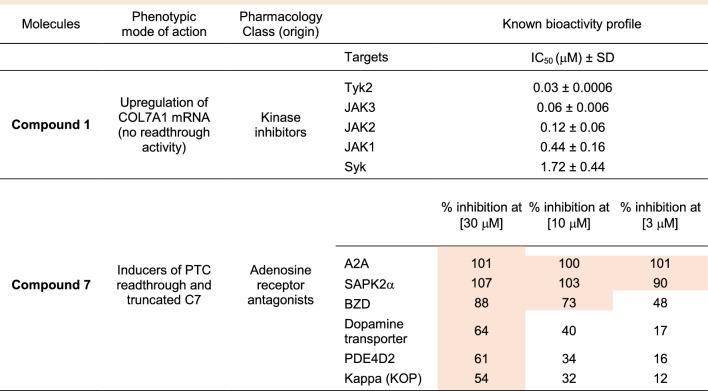
Pharmacology and selectivity profile of compound 1 and compound 7.

Known activities based on dose–response curves (IC_50_ values) or selectivity panel studies. 128 activities were evaluated in the selectivity panels: 84 kinases and 44 enzymes, GPCR, and ion channels at 3 concentrations (n = 1, duplicate determinations) (see supplementary information Tables S2 and S3). Activities showing > 50% inhibition at the indicated concentrations are highlighted in color.

Regarding the subset of microbial extracts screened, none of the known aminoglycosides included in the samples evaluated were active in the AlphaLISA assay. The aminoglycosides produced by these strains were hygromycin A/B, neomycin A, streptothricin F/D, and N-Ac-Streptothricin F, and none of them have been previously reported to be active for the modulation of C7^37^, which suggests that not all aminoglycosides have activity as upregulators of C7. On the other hand, a strain producing **chuangxinmycin** was active. **Chuangxinmycin** is an antibiotic with an indole-dihydrothiopyran heterocyclic skeleton that shows a unique antibacterial mechanism as an inhibitor of bacterial tryptophanyl tRNA synthetase (TrpRS)^41^.

### Compound 1 upregulates COL7A1 mRNA in patient cells while compound 7 and chuangxinmycin show readthrough activity in patient and recombinant cells

**Figure 3:**
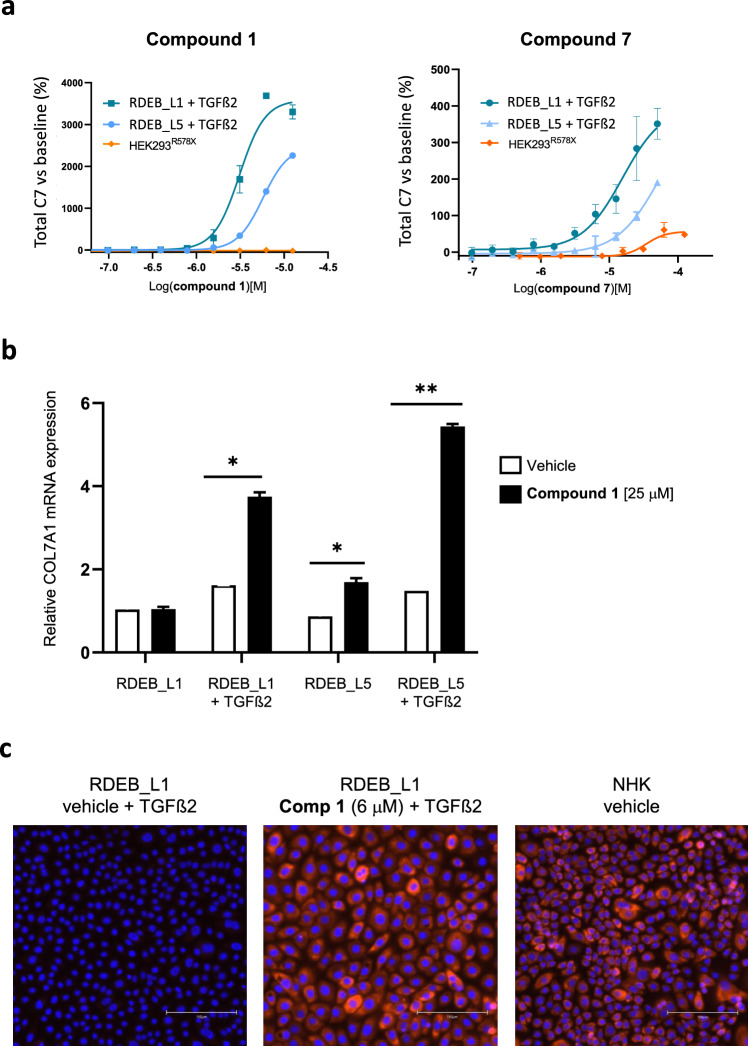
Characterization of the mode of action of representative hits. (**a**) AlphaLISA dose-response evaluation of **compound** **1** and **compound** **7** in RDEB_L1, RDEB_L5, and HEK293^R578X^ cell lines (representative curves of at least n=3 shown). The activity in RDEB_L1 and RDEB_L5 was evaluated in the presence of 20 ng/mL of TGFβ2 (**b**) Comparative qPCR analysis of the induction of COL7A1 mRNA in RDEB_L1 and L5 cells by **compound** **1** in the presence of 20 ng/mL of TGFβ2 (24h treatment). Statistically significant differences are indicated by ** p<0.001 and * p<0.05. (n=2) **c** Immunofluorescence detection of C7 in RDEB_L1 cells (truncated C7 detected by LH7.2 activity) and NHK wild type keratinocytes (full length C7) treated with vehicle or **compound** **1** and, for RDEB_L1, with 20 ng/mL of TGFβ2. Representative images are shown (n=5). Scale is indicated in each image by a white bar representing 150 μM.

Evaluation of hit compounds in patient-derived keratinocyte RDEB_L1 and HEK293^R578X^ cells indicated that, while **compound** **7** and **chuangxinmycin/3-methyl-chuangxinmycin** were active in both cell lines with different levels of efficacy and potency (Tables 1, 3), **compound** **1** was only active in the donor-derived cells RDEB_L1 and RDEB_L5 but not in the recombinant HEK293^R578X^ cell line in the AlphaLISA assay (Tables 1, 2, 3, Fig. 3a). In this cell line, the expression of mutated COL7A1 mRNA is driven by a CMV promoter, not by the natural COL7A1 promoter. This indicated that this promoter, or some other factor not present in HEK293^R578X^ cells, was required for the response to **compound** **1**, confirming the different mode of action of this compound with respect to **compound** **7** and **chuangxinmycin** series.

Assessment of the levels of COL7A1 mRNA in RDEB_L1 and RDEB_L5 cells in response to treatment with **compound** **1** showed that TGFβ2 was required for full upregulation (Fig. 3b). Treatment with **compound** **7** under those conditions did not result in an increase in C7 mRNA levels (see supplementary information Fig S5). The levels of truncated C7 resulting from **compound** **1** treatment were comparable to or even higher than those of full-length C7 in normal human keratinocytes and were readily detectable in the cytoplasm of treated cells by immunofluorescence analysis (Fig. 3c). Analysis of RDEB_L1 cells treated with **compound 1** indicated that total C7 protein could also be detected in the supernatant (see supplementary information Fig S6).

### Compound 1 and gentamicin synergize to upregulate total C7 levels

We then wondered whether a combination of **compound** **1**, lacking readthrough activity, and gentamicin as a reference readthrough drug, could have a synergistic effect. We hypothesized that increased COL7A1 mRNA levels induced by **compound** **1** could further enhance the activity of gentamicin.

**Figure 4:**
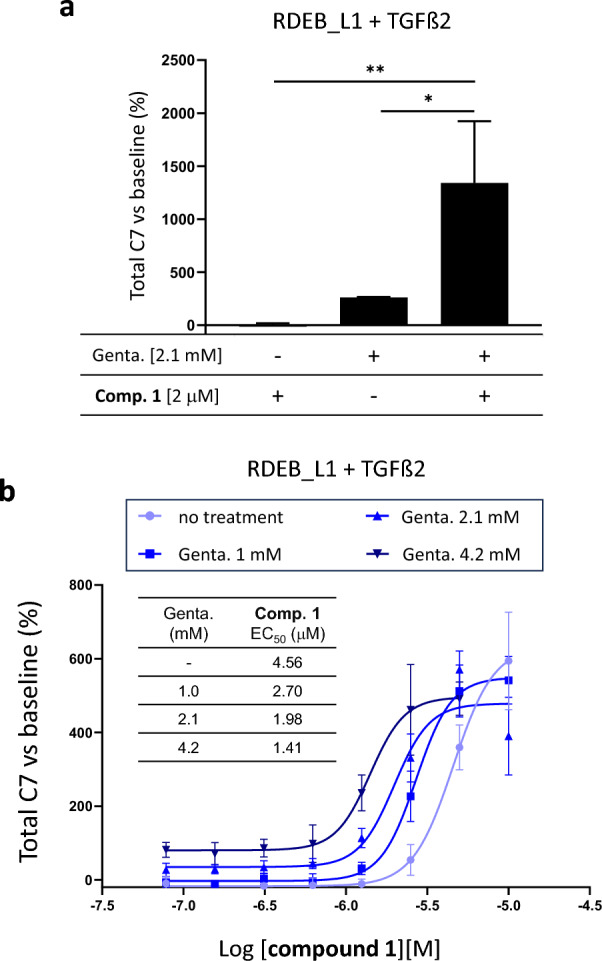
Sinergy between gentamicin and compound 1 inducing truncated C7 levels in RDEB_L1. (**a**) Synergistic effect in upregulation of total C7 levels (AlphaLISA) resulting from the combination of the indicated suboptimal concentrations of **compound** **1** and gentamicin after incubation during 48 h in the presence of 20 ng/mL of TGFβ. Statistically significant differences are indicated by **p<0.001 and *p<0.05. A representative result from n = 2 is shown. (**b**) Shift in **compound** **1** dose-response curves in RDEB_L1 cells in the presence of increasing concentrations of gentamicin. Representative curves of n=2 are shown.

A combination of **compound** **1,** and gentamicin at concentrations in the range of their EC_50_ resulted in a synergistic effect inducing a significant increase in total C7 levels detected by AlphaLISA above what would be expected for each compound individually (Fig. 4a) leading to a decrease of threefold in the EC_50_ of **compound** **1** in the presence of increasing concentrations of gentamicin (Fig. 4b). Under these conditions, no increase in the readthrough activity of gentamicin was detected in western blot (Supplementary information Fig S7).

### Compound 7 shows phenotypic selectivity for native stop codons

**Figure 5:**
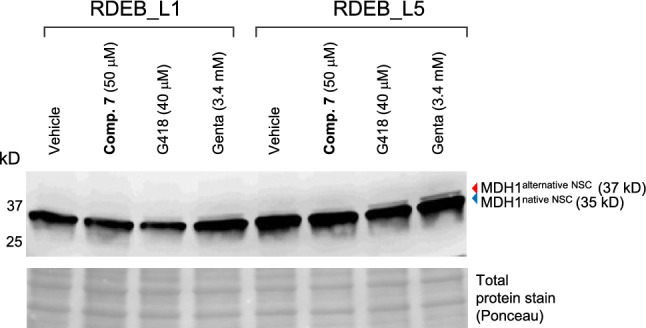
Western blot assessment of readthrough selectivity in reference MDH1 gene native stop codons (NSC). RDEB_L1 and RDEB_L5 cells were treated for 48 h with **Compound** **7** and reference compounds G418 and gentamicin at the indicated concentrations. MDH1 products resulting from the stop of translation at normal and alternative NSC are indicated (western blot is a representative result from n=2 in RDEB_L5 for **compound** **7** and G418, and one experiment each in RDEB_L1 and L5 for gentamicin). Image has been cropped for convenience (uncropped gel is presented in Supplementary **Figure** **S9**).

In the absence of a verified target or mechanism of action, a key aspect of phenotypic screening is to ensure that the phenotypic activity is sufficiently selective and has minimal or no interference in physiologically close cellular processes. In this context, the selectivity of readthrough compounds vs native stop codons (NSC) and normal codon–anticodon recognition is an important endpoint to verify the selectivity of hit compounds. For that purpose, we analyzed the translation products of MDH-1, a gene that has an alternative native stop codon that has been used as a surrogate of non-specific readthrough activity^42,43^. Analysis of MDH-1 expression in RDEB_L1 and RDEB_L5 cells by western blot showed that **compound** **7** did not induce non-specific readthrough above background levels at concentrations two and threefold above its EC_50_ in RDEB_L5 and RDEB_L1 cells respectively, while gentamicin was inducing detectable non-specific readthrough at concentrations twofold above its EC_50_ in RDEB_L1 under the same conditions (Fig. 5). No increase in detectable levels of the full-length form of C7 was evident from these experiments.

### Optimization of compound 7 selectivity profile

To further explore the potential mechanism of action underlying the readthrough activity of **compound** **7**, additional selectivity information for this compound was obtained through screening against standard selectivity panels which included 85 kinases, 36 G-protein-coupled receptors (GPCR), 5 enzymes, 3 ion channels, and 3 transporters (see supplementary information Tables S2 and S3). In these panels, c**ompound** **7** showed ≥ 50% antagonism/inhibition of A2A adenosine receptor, p38/SAPK2α kinase, and benzodiazepine receptor BZD activities at concentrations of 3 and 10 µM (Table 4), below its EC_50_ in the C7 AlphaLISA assay (Tables 1, 2, 3).

**Table 5:**
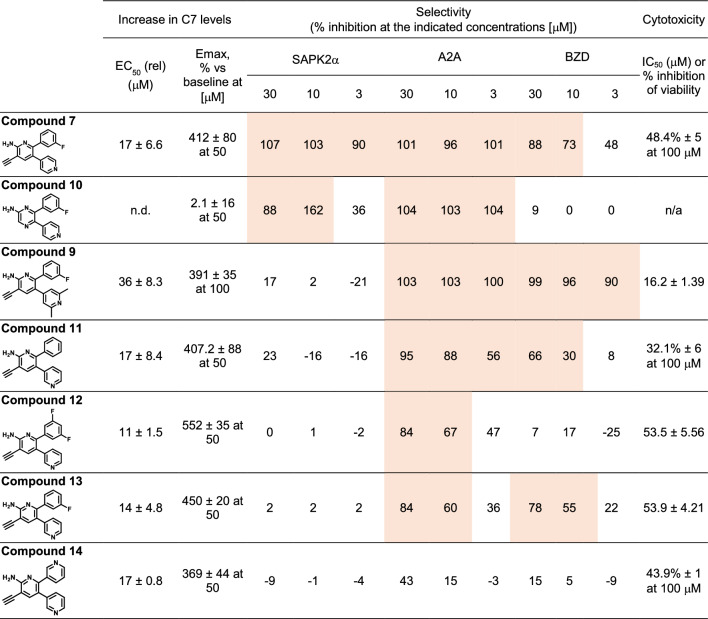
SAR analysis of the selectivity of compound 7 derivatives.

SAPK2α, A2A, and BZD activities were evaluated at 3 concentrations (n = 1, duplicate determinations) (see materials & methods for additional information). C7 Alphalisa and viability assay (cytotoxicity) values shown are from 2 separate experiments (n = 2) except for **compound** **7** (n = 14). Compound treatments in the presence of 20 ng/mL TGFß2. Activities > 50% inhibition are highlighted in color. Impact on viability assessed in CHO cell line using ATP as viability read out.

We then decided to assess whether the 3 main activities modulated by **compound** **7** (Table 4) were associated with its upregulation of C7 in the AlphaLISA assay. A limited structure–activity relationship (SAR) effort was conducted evaluating the inhibition of A2A, p38/SAPK2α, and BZD activities. Structural modifications were tested at several positions of the structure of **compound** **7** aimed at reducing activity over these targets in derivative compounds. Several derivatives showing reduced inhibition for at least one of the targets evaluated were generated, and one compound was identified, **compound** **14**, combining two of these modifications and showing improved selectivity vs the three targets while maintaining activity in the AlphaLISA assay and low cytotoxicity (Table 5). This indicates that the activity of **compound** **7** and related compounds as upregulators of C7 in the phenotypic assay is not dependent on the modulation of these targets.

### Transcriptomic profiling reveals RDEB signaling pathway signatures in patient-derived cells and a mechanism of action different from aminoglycosides

To explore the cellular pathways that could be relevant for the phenotypic activities of the hit compounds we decided to conduct RNAseq-based profiling of RDEB_L1 and NHK cells. For this study, gentamicin and G418 were evaluated as reference compounds, and **compound** **1**, **compound** **7**, **compound** **8**, **chuangxinmycin** and **3-Methyl-chuangxinmycin** were tested as representative hit compounds. All compounds were evaluated by incubating RDEB_L1 cells for 24 h in the presence of 20 ng/mL TGFβ2.

**Figure 6:**
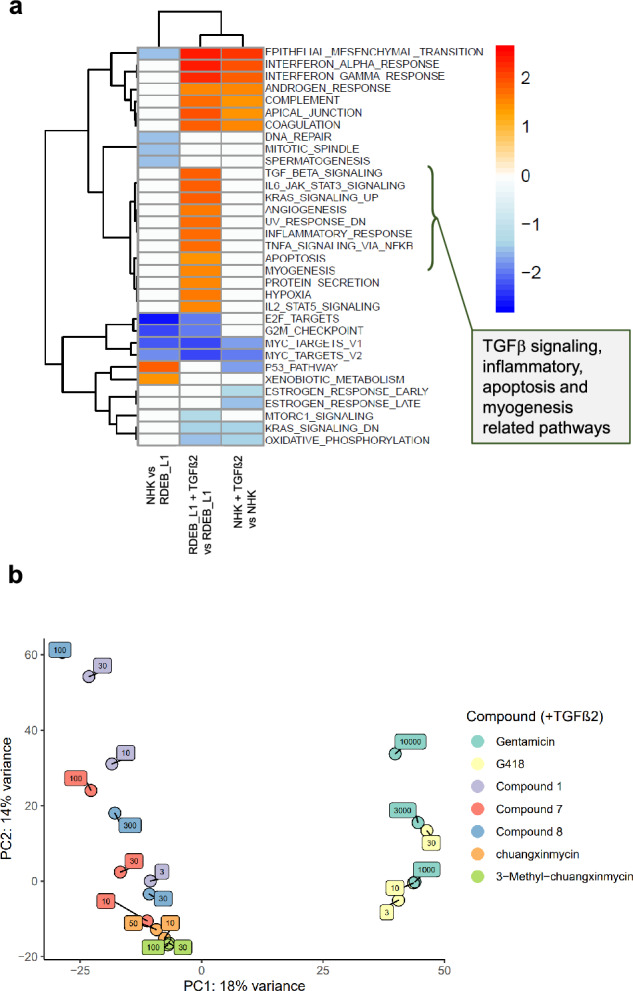
Transcriptomic profiling of RDEB_L1 and NHK cells and PCA analysis of reference compounds and representative hits. (**a**) GSEA enrichment analysis of signaling pathways in RDEB_L1 cells and normal human keratinocytes (NHK) modulated after stimulation with TGFβ2. Normalized enrichment score (NES) values are shown in the heat map. This value indicates the degree to which each pathway is overrepresented at the top or bottom of a ranked list of genes differentially expressed on each condition. Hallmarks from MSigDB showing a significant enrichment are shown in the heatmap (**b**) PCA analysis showing the differentiation between aminoglycosides and hit compounds in RDEB_L1 cells at the indicated doses in the presence of 20 ng/mL of TGFβ2 (see Materials & Methods).

Transcriptomic differences between RDEB_L1 and NHK cells, and the effect of TGFβ2 stimulation were determined using the Molecular Signatures Database (MSigDB) hallmark gene set collection as a reference for gene ontology (GO) signaling pathway analysis^44^. The transcriptomic analysis showed that several pathways related to inflammation, TGFβ signaling, apoptosis, and myogenesis were more upregulated in RDEB_L1 cells after TGFβ2 stimulation than in NHK (Fig. 6a). This was compatible with the inflammatory state, the upregulation of TGFβ, and the tissue remodeling in the absence of C7 expression that has been reported in RDEB patient keratinocytes and fibroblasts^12,36^, and indicated that RDEB_L1 cells were recapitulating the main expected disease signatures of RDEB.

The effect of the compounds at the transcriptomic level was assessed in a dose–response approach using a principal component dimensionality reduction analysis (PCA). Results showed a strong differentiation between the aminoglycosides G418 and gentamicin and the rest of the compounds evaluated (Fig. 6b).

**Figure 7:**
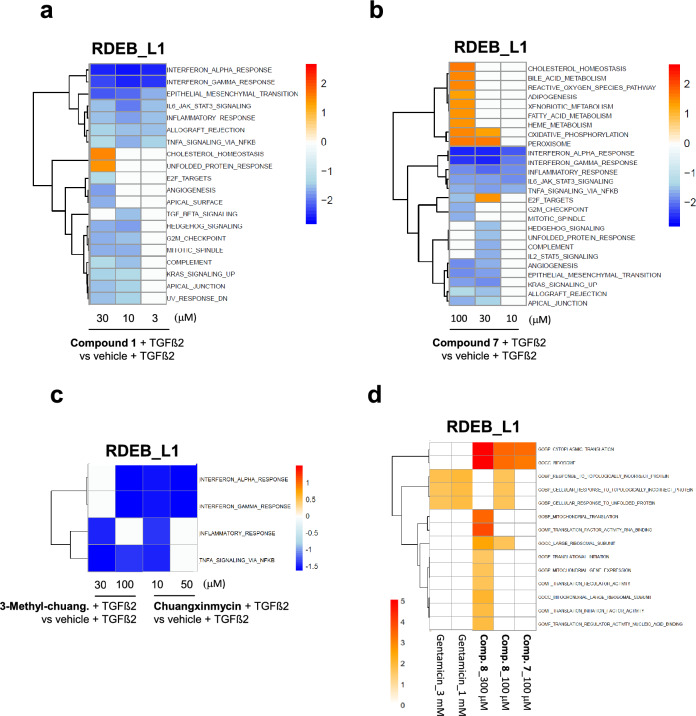
Transcriptomic profiling of the pathways modulated by representative hits in RDEB_L1 patient cells. GSEA hallmarks significant terms using (**a**) **compound** **1**, (**b**) **compound** **7**, and (**c**) **chuangxinmycin** and **3-methyl-chuangxinmycin**, in the presence of 20 ng/mL of TGFβ2**.** (**d**) GSEA enrichment values from Gene Ontology showing modulation of selected protein homeostasis and protein translation-relevant pathways by **compound** **7**, **compound** **8,** and **gentamicin**.

We then looked into the signaling pathway signatures elicited by treatment with the three representative hit families. The signature induced by treatment with **compound** **1** in RDEB_L1 cells was compatible with its pharmacology profile as a pan-JAK inhibitor and anti-inflammatory compound (Fig. 7a). Downregulation of TGFβ signaling was seen at 10 µM for **compound** **1** under conditions where truncated C7 is upregulated in these cells, in a manner that was compatible with the functional link described between C7 and TGFβ^35,36^. At the highest concentration tested (30 µM) enrichment in genes related to unfolded protein response signaling was evident under conditions where the expression of elevated levels of truncated C7 is induced (Figs. 3a, 7a). **Compound** **7** did not show any effect on the TGFβ signaling pathway. Similar to **compound** **1**, it downregulated several inflammatory pathways most likely due to its adenosine receptor pharmacology^45^, and induced upregulation of metabolism and stress-related pathways only at the highest tested concentration of 100 µM (Fig. 7b). **Chuangxinmycin** and **3-methyl**-**chuangxinmycin** showed moderate bioactivity under the conditions of the study when compared with the other 2 hit families analyzed, downregulating genes enriched in interferon, TNF, and the inflammatory response (Fig. 7c).

Lastly, a focused analysis of a selected set of gene ontology (GO) terms related to nonsense-mediated decay, translational readthrough, ribosome translation, RNA folding and translation, translation fidelity, and unfolded protein response, showed that **compound** **7** and its structurally related **compound** **8** were modulating genes specifically related to cytoplasmic translation and the ribosome in RDEB_L1 cells at the highest concentrations tested of 100 and 300 µM, while gentamicin was modulating genes related with the response to unfolded and topologically incorrect proteins (Fig. 7d). Neither **compound** **1** nor **chuangxinmycin** nor **3-methyl-chuangxinmycin** showed any impact on these pathways at any of the concentrations tested (Fig. 7d).

## Discussion

Despite recent advances offering patients a new gene-therapy-based topical treatment^26^, an unmet need still exists for the treatment of extracutaneous manifestations of RDEB. To address this need, we have used a phenotypic screening approach combining two RDEB patient-derived keratinocyte cell lines representing different *COL7A1* PTC mutation backgrounds, with a library of small molecules and microbial extracts as a source of chemical diversity. The screen was based on a gain-of-signal detection of total C7 protein. The advantage of using C7 levels as an endpoint is that it allows integrating any mode of action leading to an increase in detectable C7 protein, which could include translational readthrough activity, COL7A1 gene promoter activation (resulting from transcription factor or epigenetic modulation), and reduced degradation of C7 mRNA or protein, among other potential mechanisms.

Using this strategy, we have identified three chemical series that represent novel small molecule-based drug discovery starting points of potential application for the systemic treatment of RDEB. Two of these chemical series, represented by **compound** **7** and **chuangxinmycin**, showed translational readthrough activity while the third, represented by **compound** **1**, showed a different mode of action, upregulating COL7A1 mRNA and truncated C7 protein levels without any detectable readthrough activity.

**Compound** **1** and **compound** **7** were derived from Almirall legacy drug discovery programs aimed at identifying kinase inhibitors (compound 1 series) and adenosine receptor antagonists (compound 7 series) and their bioactivity profiles were partially characterized. However, the EC_50_ range for the C7 induction activities of these compounds was well above their previously known pharmacological activities in the nanomolar and sub-micromolar range. This could indicate that their C7 upregulating activities are disconnected from their known pharmacology or that some additional activities only evident at higher concentrations are required for their modulation of the phenotypic activity. C7 upregulation could result from the combination of several activities as a multi-target polypharmacology signature. **Compound** **7** showed remarkably low cytotoxicity (IC_50_ > 100 µM) as measured in RDEB_L1 cells and normal human keratinocytes (NHK) (Table 1, supplementary Table S1). In this context, a systematic SAR effort to explore whether the 3 top targets engaged by **compound** **7** (A2A, p38 SAPK, and BZD) were required for its C7 upregulation activity revealed that they were not necessary. This led to the synthesis of a compound with increased selectivity for these targets and still active inducing C7 in the AlphaLISA assay. This is an example of how phenotypic hits can be optimized to reduce off-target activities without compromising phenotypic activity and supports the hypothesis that C7 upregulation might not be related to the known pharmacological profile of **compound** **1** as an adenosine receptor modulator. However, more in-depth profiling would be required to confirm this.

**Chuangxinmycin** is an antibiotic discovered more than 45 years ago that has attracted the interest of medicinal chemists in the antibiotic-resistant bacteria arena^39,40^, whereas **3-methyl-chuangxinmycin** is a related compound that was recently described^40^. Like many other antibiotics, new applications such as enzyme inhibitors or antitumor drugs are continuously emerging for antibiotics, and this could be another example. No readthrough activity has been previously reported for this compound but its known mechanism of action as an inhibitor of bacterial tryptophanyl tRNA synthetase (TrpRS)^41^ may provide a starting point to decipher the molecular mechanism underlying the readthrough activity uncovered by this study. Tryptophanyl tRNA synthetase is an essential enzyme during translation both in bacteria and mammalian cells^46^.

Transcriptomic profiling of representative compounds from the three series revealed that their transcriptomic signatures were different from aminoglycosides at concentrations relevant for their C7 upregulation or translational readthrough activities. Interestingly, a focused GO analysis exploring terms related to the biology of protein translation revealed that hits from **compound** **7** series were mobilizing genes associated with cytoplasmic translation and ribosome, GO terms that were not related to those mobilized by aminoglycosides in this dataset and which were not related to genes modulated by **compound** **1** either.

**Compound** **1** increases levels of truncated but not full-length C7 in donor-derived cells indicating the absence of any readthrough activity. This upregulation correlates with the induction of COL7A1 mRNA. Based on its known pharmacology, we could speculate that the induction of truncated C7 expression by **compound** **1** could be explained by its downregulation of the JAK/STAT pathway, which would relieve the blockade of SMAD proteins by STAT3, allowing TGFβ-mediated upregulation of the COL7A1 gene promoter by SMAD transcription factors^47,48^. Upregulation of JAK-STAT signaling pathways in RDEB skin has been reported as a main hallmark of RDEB lesions^49^. In a CRISPR screen evaluating pathways involved in C7 upregulation the anti-inflammatory flavonoid kaempferol, a reported inhibitor of JAK3, was identified as an inducer of COL7A1 mRNA^31,50,51^.

Upregulation of COL7A1 mRNA could have the potential to enhance the efficacy of other RDEB therapies and increase the basal readthrough levels seen in some RDEB patients. Interestingly, our results indicate that, in addition to their readthrough activity, aminoglycosides G418 and gentamicin also induce the upregulation of truncated C7, likely due to some indirect mechanisms controlling gene expression in a promoter or epigenetic mechanism-dependent manner^52^, or through inhibition of NMD-mediated degradation of mutated C7 mRNA^30^. We hypothesize that, in addition to translational readthrough, upregulation of truncated C7 expression could contribute to the efficacy of aminoglycosides. In this context, a potential synergistic combination of the mode of action represented by **compound** **1** with aminoglycosides could provide an opportunity to optimize their efficacy improving their safety/efficacy ratio.

In addition to the potential benefit that increased levels of COL7A1 mRNA containing nonsense mutations could have in the context of translational readthrough, there is rationale to support the potential beneficial effect that increased expression of truncated forms of C7 protein could have in RDEB, regardless of the type of mutation causing it. The N-terminal NC1 domain of C7 contains a fibronectin-binding region and could provide partial structural or functional integrity relief in the absence of fully functional anchoring fibrils. Analyses of the binding features of the NC1 domain demonstrated its affinity to laminin 5 and collagen IV (K_a_ 48 nM and 36 nM respectively)^53^. In addition, evidence obtained in an RDEB mouse model by Ito et al., where expression of truncated forms of C7 improved survival^54^, supports the concept of truncated forms of C7 with partial structural or functional properties whose upregulation could result in some clinical benefit in RDEB.

TGFβ2 upregulates expression of C7 in keratinocytes and TGFβ2 signaling is increased in RDEB lesions under conditions of reduced C7 expression^36^, suggesting a mutual autoregulatory loop that is disrupted in RDEB. The presence of TGFβ2 in our in vitro model of RDEB offered an opportunity to better replicate the physiological conditions of RDEB lesions and the homeostatic regulatory interactions that take place between C7 and TGFβ. From a functional perspective, we could speculate that some forms of truncated C7 could still down-modulate TGFβ2 signaling, reducing fibrosis and the risk of SCC while improving re-epithelization and wound healing. An earlier report suggesting that expression of truncated C7 could be associated with an increased risk of developing squamous cell carcinoma in RDEB patients^55^ has not been confirmed in subsequent studies looking at the origin of SCC in RDEB^56,57^.

In this context, it is becoming increasingly clear that the deficiencies caused by the absence of C7 reach beyond its structural function in dermis/epidermis anchoring fibrils^58^, and also result from the disruption of a network of functional interactions with roles in extracellular matrix protein secretion and homeostasis^59^, fibrosis, inflammation and cellular transformation. Results obtained in RDEB monozygotic twins and siblings show that the levels of C7 in the skin do not directly correlate with disease severity^12,60^. Fibroblasts from the twin with more severe clinical manifestation showed increased levels of TGFβ1 and pro-inflammatory cytokines IL-6 and MCP-1, constitutive TGFβ signaling activation leading to increased p-Smad2, p-p38, and p-ERK1/2, and downregulation of decorin, a proteoglycan reported to prevent disease progression in a murine model of RDEB^61^.

The emerging concept is that RDEB pathophysiology triggered by the absence or deficiency in C7 function is not only associated with the disrupted physical scaffolding role of C7 in anchoring fibrils but also integrates inflammatory and pro-fibrotic components that ultimately shape the clinical phenotype of each patient and are influenced by additional genetic variability. This could explain the apparent discrepancy between the severity of the RDEB and the level of expression of full-length C7 and offers an opportunity to take advantage of non-structural functions of C7 that could also be contributed, at least in part, by increased levels of truncated forms of this protein.

One limitation of this study is that, in the absence of evidence obtained in in vivo models or reconstituted RDEB patient-derived skin, it does not provide definitive proof that the C7 induced or upregulated by the hit molecules identified is fully functional. However, Cogan et al.^37^, showed that, for a variety of nonsense mutations found in RDEB patients, including the R578X mutation used in this work, the full length collagen induced by aminoglycosides was forming stable triple helices. This evidence supports the hypothesis that the full length C7 detected after treating HEK293^R578X^ cells with the molecules identified in this study could result in the formation of functional C7. In any case, the chemical series that we have identified represent a solid starting point for future work aimed at demonstrating full functionality of the upregulated C7.

In conclusion, this work has provided three new chemical series representing chemistry and biology which, upon further optimization and functional characterization, could be of potential application for the systemic treatment of RDEB. **Compound** **7** and **chuangxinmycin** chemical series show readthrough activities while **compound** **1** induces expression of truncated C7 and can be combined with gentamicin to further enhance the activity of both molecules to upregulate truncated C7. These results offer an opportunity to explore novel systemic strategies for the treatment of RDEB that could complement topical therapies to address the remaining unmet needs of patients suffering from this devastating disease.

## Materials and methods

### Compounds and in silico compound selection

Source of reference compounds: Gentamicin (G1914, SIGMA), G418 (A1720, SIGMA), amlexanox (SML0517, SIGMA), caffeine (C0750, SIGMA), ataluren (14,832, CAYMAN).

Small molecule compound library: 8,273 compounds from Almirall compound library were tested in the primary screen. This subset was the result of merging (a) 5,000 compounds derived from legacy in-house drug discovery projects containing a subset of 200 approved marketed drugs, with (b) 3,435 compounds bearing structural similarity with the following reference readthrough and anti-NMD molecules (also included in the selection): aescin^62^, amlexanox^63^ atalurem^23^, caffeine^64^, gentamicin^14^, oxibendazole^62^ and puromycin^65^. Compounds were selected based on physicochemical properties (molecular weight between 300 and 650 Da), lack of structural alerts (reactive and toxic groups), similarity to readthrough and anti-NMD molecules calculated considering 4 different fingerprints: ECFP4, FCFP6, FCFC6, and MDL keys and Tanimoto similarity coefficient, and diversity, calculated from FCFP4 fingerprint distances.

Microbial extracts library: a subset of MEDINA’s Microbial Natural Products (NP) Library of 20,160 extracts containing 10,560 actinomycetes and 9,600 fungal extracts was used. For actinomycetes, 33 extracts derived from 12 strains that were known producers of aminoglycoside compounds were included to ensure that known mechanisms of action were represented. Actinomycetes extracts were obtained from strains isolated from samples collected from more than 26 different countries and 70% were taxonomically identified as belonging to 37 genera from 13 families. Fungi extracts were representative of basidiomycetes (27%), endophytes (30%), lichen-associated fungi (7%), rhizosphere fungi (23%), and high-diversity fungi (13%) from several ecosystems (leaves, soil, sediments, coprophilous). For fungal, extracts were derived from strains isolated from samples collected in more than 14 countries with 44% of strains identified including more than 41 taxonomical orders. MEDINA’s Microbial Natural Product Extracts Library was prepared by extraction of culture broths with acetone (1:1) and solvent evaporation of supernatants in the presence of DMSO 20% to 2XWBE (Whole Broth Equivalent).

### Normal human keratinocytes and RDEB patient-derived and HEK293 cell lines

Patient-derived transformed cell lines were generated from keratinocytes isolated from surplus cell-biobank tissue obtained from patient skin samples harvested for molecular diagnosis after ethical committee approval (reference HULP: PI-3911, Hospital Universitario de la Paz, Madrid, Spain) and patient’s informed consent. Samples, experimental procedures, and all associated data and results were treated in compliance with local ethical and data protection regulations and in agreement with the proposal approved by the Ethical Committee. Skin keratinocytes were isolated as previously described^66^. Immortalization resulted from the forced expression of human papillomavirus HPV16 E6/E7 antigens^66^. For transformation, retroviral particles obtained from the supernatant of HEK293T cells were transfected with pLXSN16E6E7, pNG-VL3-MLV-gag-pol, and pNGVL3-4070 plasmids^66–68^. After retroviral infection, the cells were kept in KGM medium in the presence of a feeder layer and were amplified during several passages at a ratio of 1:4 in each passage. After 18 passages, the keratinocytes were cultured in the absence of a feeder layer in the KGM medium.

For cell culture maintenance and AlphaLISA and western blot assays, RDEB patient-derived cells and human adult epidermal keratinocytes (Lonza 00192627) were grown in complete KGM™ Gold™ (KBM™ Gold™ Basal Medium with SingleQuots™) excluding the GA-100, (Cultek H3192060). For cell maintenance, the medium was removed, and cells were rinsed with Hepes Buffered Saline Solution (Lonza CC-5024). After aspirating the HEPES, TripLE express solution (Gibco 12604054) was added to detach the cells. For neutralizing the TripLE express, Trypsin Neutralizing Solution (Lonza CC-5002) was added and then the cell suspension was centrifuged at 140 g for 5 min at room temperature, the supernatant was discarded, and the pellet was resuspended in KGM complete medium without GA-100 at the desired density.

For the generation of the HEK293^R578X^ recombinant cell line, site-directed mutagenesis was used to generate a mutated COL7A1 cDNA containing a C to T change at nucleotide 1732, resulting in a change in Arg578 to a UAG termination codon. The resulting mutated COL7A1 cDNA was then stably transfected into HEK293 cells after verifying the absence of expression of COL7A1 in this cell line. Zeocin was used as a selection antibiotic to obtain the stably transfected clones which were selected by limiting dilution.

HEK293^R578X^ was cultured in DMEM high glucose supplemented with 10% FBS and 2 mM Glutamax (Thermo Fisher 61965026). PBS was used for the rinse step and TripLE express was used to detach the cells. DMEM complete media was used for neutralizing the TripLE express and, after centrifugation at 140 g for 5 min at room temperature, the supernatant was discarded, and the cells were resuspended in DMEM complete media at working densities.

### AlphaLISA assay and additional biochemical assays

The AlphaLISA assay was developed in-house by using the biotin-conjugated rabbit polyclonal antibody anti-C7 (LSBio LS-298147) and conjugating the acceptor beads (PerkinElmer 6772001) to a monoclonal anti-Collagen type VII antibody produced in mouse, clone LH7.2 (Thermo MA5-13,432). Formulation of the mAb clone LH7.2 was exchanged to PBS and concentrated 5 × by using Amicon® Ultra-0.5 centrifugal filters up to 1 mg/ml. The conjugation procedure was carried out according to the protocol provided by PerkinElmer.

For compound treatment in RDEB patient-derived transformed keratinocytes, 5,000 cells were plated in 20 µL of supplemented KGM media containing 20 ng/mL TGFβ2 (SIGMA GF113) without GA-1000 in 384-well cell culture plates (Greiner 781098). The day after, 5 µL of test compounds prepared 5 × in supplemented KGM without GA-1000 were added over cells and incubated for 48 h at 37 °C and 5% CO_2_. For compound treatment in HEK293^R578X^, 10,000 cells were plated in 20 μL of DMEM (High Glucose, 2 mM L-Glutamine, sodium pyruvate, and FBS 10%) in 384-well cell culture plates (Greiner 781,098,). The day after, 5 μL of test compounds prepared 5 × in DMEM complete medium were added over cells and incubated for 48 h at 37 °C and 5% CO2.

After the treatment process, 2.5 µL of RIPA buffer 10 x (Abcam ab156034) were added over the 25 ml of cells for 20 min at room temperature to obtain a complete cell lysis. Then, 2 μL of sample (i.e., cell lysate with supernatant) were transferred to the AlphaLISA plate (PerkinElmer 6,059,340) containing 8 ml of antibodies mixture (5 mg/ml of acceptor Beads-Ab and 0.75 mg/ml of biotin-rAb) prepared in AlphaLISA buffer 1 x (PerkinElmer AL00F). The reaction was incubated for 90 min at room temperature. Finally, 10 μL of 80 mg/ml of donor beads (PerkinElmer 6760002,) were added to the reaction under subdued light, mixed gently, and incubated for 3 h at room temperature. AlphaLISA signal was detected with an Envision microplate reader (Perkin Elmer) using the following setting: Filter: AlphaScreen, Excitation time: 180 ms, Integration time: 300 ms.

Enzymatic Syk kinase activity assays were performed as previously described^69^. Enzymatic activity of JAK1, JAK2, JAK3, and Tyk kinases was evaluated as previously described^70^.

### High throughput screening (HTS) of small molecules and microbial extracts

A primary screening with the 8,273 small molecules subset from Almirall compound library and 20,160 microbial extracts from MEDINA’s library was conducted on RDEB_L1 and RDEB_L5 cell lines by AlphaLISA assay. The assay was carried out following the protocol described above optimizing the automatization of plating the cells by using a Multidrop Combi (Thermofisher), a Freedom EVO (Tecan) liquid handler robot for sample distribution, and the AlphaLISA procedure with a Multidrop Combi. Batches of eight 384-well plates were processed at the same time to save reagents and disposable labware. Before the AlphaLISA, the viability of the treated cells was verified by brightfield bioimaging using the Operetta CLS High Content Analysis System (PerkinElmer). All the plates assayed presented 16 wells of negative controls (vehicle, DMSO) and 16 wells of positive controls (5 mg/ml gentamicin). A pilot run was performed testing the small molecules subset at 25, 10, 5, and 1 μM concentrations and the microbial extracts subset at 1:20, 1:40, 1:100, and 1:200 dilutions, and the most concentrated option was selected in both cases to perform the screening as cytotoxicity was not detected in any of them. The final concentration for the HTS was 25 μM for small molecules and a dilution of 1:20 for the microbial extracts. The quality control of the screening was assessed by Z’-factor (> 0.55) and Signal Window (> 6) for all the plates tested. Z'-Factor and signal window are quality control parameters that reflect the robustness and reproducibility of the assay. Z'-Factor is a statistical parameter used to evaluate the quality of an assay and it is calculated by relating the sum of the standard deviations for the reference wells to the signal range given by the difference in their mean values. Signal Window (SW) is the ratio of the median value of the raw data for positive and negative controls. The 193 most active small molecule hits and the 62 most active microbial extracts were selected. These active samples were cherry-picked and assayed for assay interference using the AlphaLISA without cells. After activity and assay interference verification and dose–response evaluation, several small molecules representing 2 chemical series and 2 natural products derived from 1 microbial extract were finally selected as hits.

### Identification and isolation of compounds from the microbial extract hit

A 3 L scaled-up broth of Fundación MEDINA producer strain (an actinobacteria from the *Streptomycetaceae* family, isolated from the soil rhizosphere associated with a *thymus* sp. collected in El Margen, Granada), was generated in the same fermentation conditions that resulted active in the HTS campaign (KHC medium, 28 °C, 6 days) in 500 mL flasks with 150 mL of broth each and was extracted by adding an equal volume of acetone (3 L) and shaking at 220 rpm for 2 h in flasks. The extracted mycelium was separated by filtration in a Büchner, and the remaining acetone/water mixture extract was concentrated under heated nitrogen steam to the initial fermentation volume (3 L). This aqueous residue was loaded into an HP-20 resin chromatographic column (65 g, 32 × 120 mm) and eluted with a linear gradient of 5 to 100% of acetone in water for 35 min at 10 mL/min followed by a 100% acetone washing step for 10 min, to yield 25 fractions. A 1/20 aliquot of each fraction was dried in a vacuum centrifuge and dissolved in 20% DMSO/ 80% water and evaluated for activity. Among the active fractions (FL006 to 12), two were selected as more chemically representative according to their complexity by analytical C-18 reverse phase HPLC at 210 nm. Next, 30 mg of each one were dissolved in 100% DMSO and subjected to preparative reversed-phase HPLC in a Zorbax SB-C8 column (21.2 × 250 mm, 7 μm; 20 mL/min; UV detection at 210 and 280 nm) applying a linear gradient of acetonitrile in water from 5 to 33% for 35 min at 10 mL/min, followed by a 100% acetonitrile washing step for 10 min. Fractions were collected every 0.5 min, dried in a vacuum centrifuge, and dissolved in 20% DMSO / 80% water to be evaluated for activity. Two correlative active groups of fractions could be determined, a common one in retention time to both fractionations at 12–14 min, and an exclusive one for the FL008 fraction at an earlier retention time of 7–8 min. Both chromatographic regions were analyzed by High-Resolution Mass Spectrometry and NMR spectroscopy, and the identity of the bioactive compounds was established as chuangxinmycin (3.0 mg) and 3-methyl-chuangxinmycin (0.2 mg) (**Fig.** 2**a**).

### Cell cytotoxicity and viability

The viability of RDEB patient-derived cell lines or NHK was evaluated by measuring the ATP levels as an indirect readout of the potential cytotoxicity effect of compounds. Cells were plated and treated exactly with the same conditions as those followed in the AlphaLISA assay, described above. To measure ATP levels, cells were processed using the ATPlite™ 1step luminescence-based assay (PerkinElmer 6,016,731), following the manufacturer’s protocol. 25 μL of ATPlite™ reagent was added directly over cells and plates were covered with a sealing film and shaken for 25 min using a microtiter plate shaker. Luminescence was measured in a Luminoskan Ascent luminometer (Thermo Fisher Scientific). 10 μM of Staurosporine was used as a reference compound and control for the total cytotoxicity.

### Immunofluorescence

RDEB patient transformed keratinocytes or normal human keratinocytes (NHK) were plated in a 96-well High Content Imaging microplate (Corning 4517) at 10,000 cells per well in 80 μL of supplemented KGM media without GA-1000, the media used for RDEB also was supplemented with 20 ng/mL of TGFβ2, and the cells were incubated at 37 °C and 5% CO_2_. The day after, 20 µL of test compounds prepared 5 × in supplemented KGM without GA-100 were added and incubated for 48 h at 37 °C and 5% CO2.

For the immunofluorescence analysis of C7, cells were permeabilized by aspirating the media and adding 50 µl of 100% ice-cold methanol for 15 min at 4 °C. Afterwards, cells were washed three times in PBS and a blocking step was carried out by adding 100 μL of blocking buffer (PBS, 5% serum goat and 0.3% Triton X-100) and incubating 1 h at room temperature. Blocking buffer was aspirated and 50 µL of 3 µg/mL of primary antibody anti-collagen type VII (SIGMA HPA042420) diluted in diluent buffer (PBS, 1% BSA, and 0.3% Triton X-100) was added and incubated for 2 h at room temperature. After washing the cells three times in PBS, 50 µL of 10 µg/mL of goat anti-rabbit IgG secondary antibody, Alexa Fluor™ 555 conjugated (A-21428, Thermo) prepared in diluent buffer were added and incubated for 1 h at room temperature. For the nuclei staining, 5.5 µL of a 10 × solution of Hoechst 33342 prepared in PBS was added and was incubated during the last 15 min of secondary antibody incubation. Lastly, cells were washed with PBS, leaving 50 µL of PBS in the plate. Images were taken in an EVOS M7000 multi-channel fluorescence and transmitted light imaging system and the imaging analysis was performed using the CELLeste software. Collagen VII was monitored by analyzing the total region intensity red and cell counting was monitored by object counting in the DAPI channel.

### Real-time quantitative PCR

For real-time qPCR, 15,000 RDEB keratinocytes or normal human epidermal keratinocytes were plated in a 96-well cell culture plate in 80 µL of supplemented KGM medium without GA-1000 and containing 20 ng/mL of TGFβ2 for RDEB keratinocytes, and cells were incubated overnight at 37 °C and 5%CO_2_. The day after, cells were treated with 20 µL of test compounds prepared 5 × in supplemented KGM without GA-100, for 24 h at 37 °C and 5% CO_2_. For the mRNA extraction, the medium was removed, cells were washed with PBS, and conserved at -80 °C until the total RNA extraction.

RNA extraction was conducted using the RNeasy 96 Kit (4) (Qiagen 74181) following the manufacturer’s instructions. Total RNA was recovered in 50 µL of RNase-free water and the yield of extracted RNA was quantified using a NanoDrop 2000 Spectrophotometer (ThermoFisher). Purified RNA was stored at -80 °C until RT-qPCR.

Real time-qPCR was performed by using the iTaq Universal Probes One-Step Kit, (cat. 1725140, Bio-Rad) and according to the manufacturer’s instructions. RT qPCRs were run in QuantStudio™ Real-Time PCR System (Applied Biosystems) or in a CFX96 Touch Real-Time PCR Detection System (BioRad) for 384 or 96 well format, respectively. For the synthesis of cDNA qPCR reactions, COL7A1 (Hs00164310_m1) or a GAPDH (Hs02758991_g1) TaqMan™ probe (Thermo Fisher Scientific) was used. RT-qPCR was performed to quantify the COL7A1 mRNA (exon 64) expression.

For COL7A1 quantitative gene expression, human GAPDH was used as the endogenous control. Results were analyzed with the QuantStudio Real-Time PCR Software.

### Western blot

For western blot, RDEB or normal human keratinocytes were lysed in protein extraction buffer (50 mM Tris–HCl [pH 7.5], 100 mM NaCl, 1% Nonidet P-40, and 4 mM EDTA) containing proteinase inhibitors cocktail (Complete Mini, EDTA free; Roche Diagnostics, Mannheim, Germany). Lysates were incubated for 30 min on ice and centrifuged at 15,000 g for 30 min at 4 °C. Supernatants were then collected and concentrated by ultrafiltration using Amicon Ultra columns (10 kDa; Millipore, Ireland). Protein concentrations were measured using the Bradford assay (Bio-Rad, Hercules, CA). For each sample, 40 mg total protein was resolved on NuPAGE Novex 3%–8% Tris–Acetate gel electrophoresis (Invitrogen, Carlsbad, CA) and electrotransferred to nitrocellulose membranes (Invitrogen, Carlsbad, CA). For type VII collagen analysis, blots were probed with a monospecific polyclonal anti-C7 antibody (a generous gift from Dr. A. Nystrom, University of Freiburg). Invitrogen anti-MDH1 polyclonal antibody (PA550446, 1:1000) was used to detect MDH1. Standard antibodies against a reference protein were used as a loading control, as indicated. Visualization of the resulting signal was performed by incubating with horseradish peroxidase (HRP)- conjugated anti-rabbit antibody (Amersham, Burlington, MA) and West Pico Chemiluminescent Substrate (Pierce, Rockford, IL).

### Selectivity panels and RNAseq transcriptomic hit profiling

Selectivity was evaluated at Eurofins Pharma Discovery Services at 3 µM, 10 µM, and 30 µM in duplicate using a selection of 84 representative kinases and 44 GPCRs, transporters, nuclear receptors (NR), enzymes, and ion channels^71^.

The RNAseq (HTPathwaySeq®) analysis was conducted at CellCarta (Antwerp, Belgium) using samples provided by Almirall. A total of 376 cell lysate samples representing 4 biological replicates of 94 samples were provided to CellCarta in four 96-well plates. On each 96-well plate, two empty wells were allocated to include CellCarta’s reference samples. Samples from the same biological group were distributed over the different plates to not confound inter-plate variability with biological signal. For this study, gentamicin (1, 3, 10 mM) and G418 (3, 10, 30 µM) were evaluated as general reference compounds, while **compound** **1** (3, 10, 30 µM), **compound** **7** (10, 30, 100 µM), **compound** **8** (30, 100, 300 µM), **chuangxinmycin** (10, 50 µM) and **3-Methyl-chuangxinmycin** (30, 100 µM) were tested as representative hit compounds. The compounds were evaluated in RDEB_L1 cells in the presence of 20 ng/mL TGFβ2 to mimic RDEB physiology and in normal human keratinocytes with no TGFβ2 treatment. Cells were treated during 24 h. Controls for RDEB_L1 cells not treated with TGFβ2 and RDEB_L1 and NHK cells treated with DMSO in the absence of compounds were also included. The conditions of the assay were identical to those described for the Real-time quantitative RT-PCR (see above).

For the HTPathwaySeq, over 460 M reads were generated for 376 samples of interest, resulting in about 1.22 M reads per sample on average. HTPathwaySeq is based on a 3’-end library preparation method, and gene body read coverage is concentrated towards the 3’-end of the genes. For quality control, a selection of lysate samples was checked for gDNA contamination and RNA content using qPCR before library preparation. RNA samples were processed to generate RNA-sequencing libraries using CellCarta’s HTPathwaySeq procedure based on the QuantSeq (Lexogen) 3’-end library preparation workflow. The quality of a selection of samples was assessed with the 2100 Bioanalyzer instrument (Agilent). Libraries were quantified using qPCR and pooled for single-end sequencing on a NextSeq 500 (Illumina) according to CellCarta’s standard operating procedure. Raw reads were mapped to the human genome (hg38) and gene expression was quantified based on the Ensembl transcriptome annotation (version 85) using HTSseq. Raw counts were normalized using DESseq2 prior to differential gene expression analysis. For each cell line, samples were clustered based on log-transformed normalized counts using tSNE. A perplexity parameter of 35 and a maximum number of 1000 iterations were applied. Genes with more than 10 normalized counts in at least 3 samples were used as input. Analysis was run in R (v3.2) with a fixed seed. To identify differential pathways enriched among the up or downregulated genes of a given condition, we performed Gene Set Enrichment analysis using the hallmarks gene set collection from the Molecular Signature Database (v6.2) and Gene Ontology terms, applying 1000 permutations. For each contrast, genes ranked by fold change were used as input. PCA was applied on the fold changes of each compound vs their associated vehicle using the 500 genes with the greatest variance. Arrows connect the different incremental dosages used on each compound to observe their differentiation patterns. Pathway enrichment and PCA were performed using R v.3.2. Pathway enrichment was analyzed with cluster profile package. PCA was applied on the fold changes of each compound vs their associated vehicle using the 500 genes with the greatest variance. Arrows connect the different incremental dosages used on each compound to observe their differentiation patterns.

The procedure followed for the cell treatments of RDEB-L1 and NHEK, was the same as that used in the RT-qPCR study. Cell lysis and sample preparation were conducted according to CellCarta’s instructions: Plates of treated cells were centrifuged for 5 min at 600 g at 4 °C, and the medium was carefully removed to avoid disturbing the cells. Then, cells were washed in PBS, centrifuged and the PBS was removed again. Immediately, 50 µL of cell lysis solution mix provided by CellCarta (HTPathwaySeq cell lysis buffer, DNase, and proteinase K) were added over cells and incubated for 10 min at room temperature. After checking that cells were lysed, the samples were transferred to 96-well PCR plates, and then sealed and stored at -80 °C to be sent to CellCarta.

### Statistical analysis

Statistical analyses were performed using GraphPad Prism v. 7.04 (La Jolla, California). Results are shown as mean ± SD of experimental replicates. Dose–response curves to assess compound treatment effect were plotted in a four-parameter logistic model. Relative EC50, representing the total drug concentration producing a 50% effect, was calculated in the four-parameter logistic equation. Comparisons of collagen protein and mRNA expression in different experimental conditions were made using ANOVA, followed by a post hoc test. statistical significance was considered with p values < 0.05.

### Supplementary Information

Supplementary Information.

## Data Availability

Transcriptomic datasets and unique biological samples used in this study are available to qualified academic researchers upon a substantiated request that aligns with the objectives specified in the participant’s consent and project ethics approval. The transfer of biological samples will be subject to a material transfer agreement. All other datasets used and analyzed in this manuscript and not included in supplementary information are available from the corresponding authors on reasonable request.
