# Supplementary material for: Identification of novel small molecule-based strategies of COL7A1 upregulation and readthrough activity for the treatment of recessive dystrophic epidermolysis bullosa
Source: Sci Rep. 2024 Aug 16;14:18969. doi: 10.1038/s41598-024-67398-8 (PMC11329504; doi:10.1038/s41598-024-67398-8)
Supplement: Supplementary file 1 — Supplementary Information. [file 41598_2024_67398_MOESM1_ESM.docx]

**Supplementary information**

**Identification of novel small molecule-based strategies of COL7A1 upregulation and readthrough activity for the treatment of recessive dystrophic epidermolysis bullosa**

Irene Jover^1^, Maria C. Ramos^2^, María José Escámez^3,4,5,6^, Estrella Lozoya^1^, José R. Tormo^2^, Diana de Prado-Verdún^6^, Ángeles Mencía^3,4,5,6^, Mercè Pont^1^, Carles Puig^1^, Marie-Helene Larraufie^1^, Cristina Gutiérrez-Caballero^1^, Fernando Reyes^2^, Juan Luis Trincado^1^, Vicente García-González^1^, Rosario Cerrato^1^, Miriam Andrés^1^, Maribel Crespo^1^, Francisca Vicente^2^, Nuria Godessart^1^, Olga Genilloud^2^, Fernando Larcher^3,4,5,6,7^ and Arsenio Nueda^1,7^

^1^ R&D Centre, Almirall S.A., Laureà Miró 408-410, 08980 Sant Feliu de Llobregat, Barcelona, Spain.

^2^ Fundación MEDINA, Av. Conocimiento 34, Parque Tecnológico de la Salud, 18016 Granada, Spain.

^3^ Universidad Carlos III de Madrid (UC3M), Madrid, Spain.

^4^ U714-CIBER de Enfermedades Raras (CIBERER-ISCIII).

^5^ Instituto de Investigación Sanitaria, Fundación Jiménez Díaz (IISFJD), Madrid, Spain.

^6^ Centro de Investigaciones Energéticas, Medioambientales y Tecnológicas (CIEMAT), Madrid, Spain

^7^ Corresponding authors: [arsenio.nueda@almirall.com](mailto:arsenio.nueda@almirall.com), [fernando.larcher@ciemat.es](mailto:fernando.larcher@ciemat.es)

**
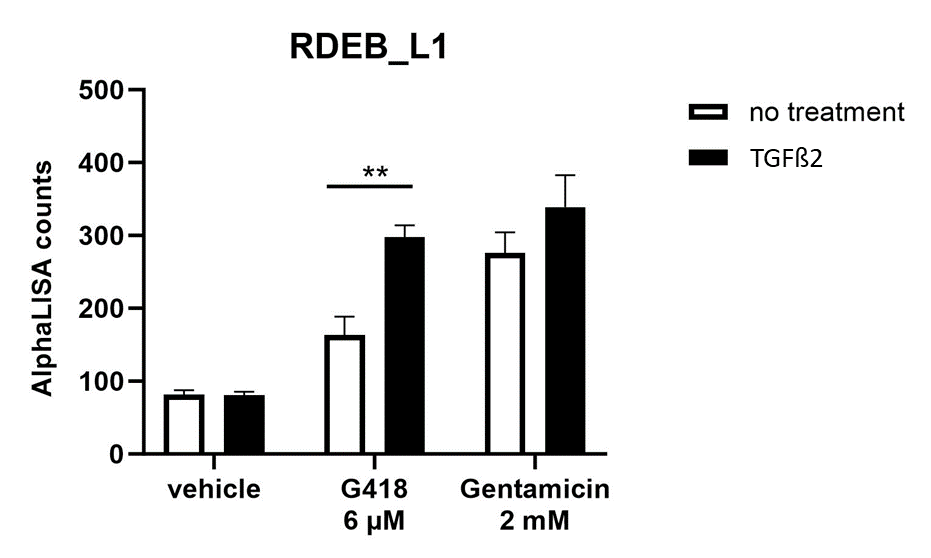
**

**Figure S1** AlphaLISA quantification of total C7 protein in total lysates of RDEB_L1 cells after treatment with vehicle, G418 or Gentamicin during 48 h, with or without 20 ng/mL of TGFß2.


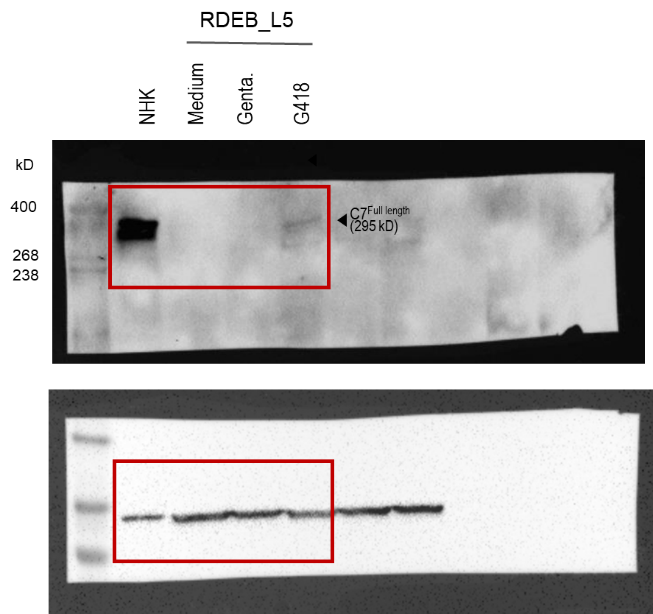
**
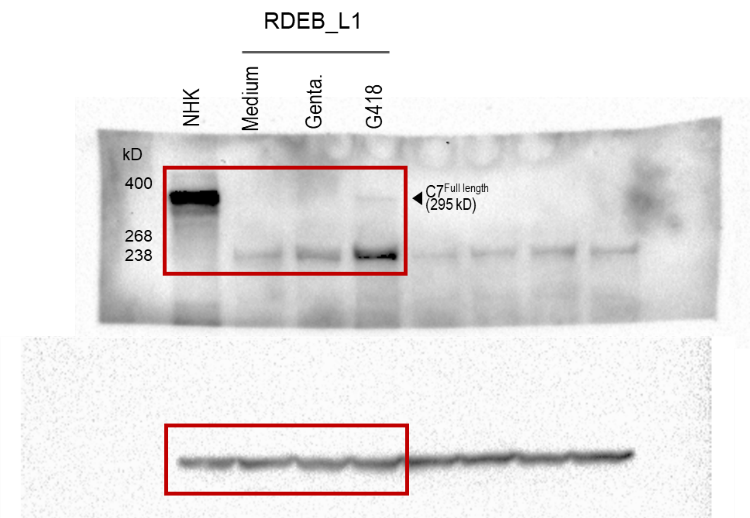
**

**
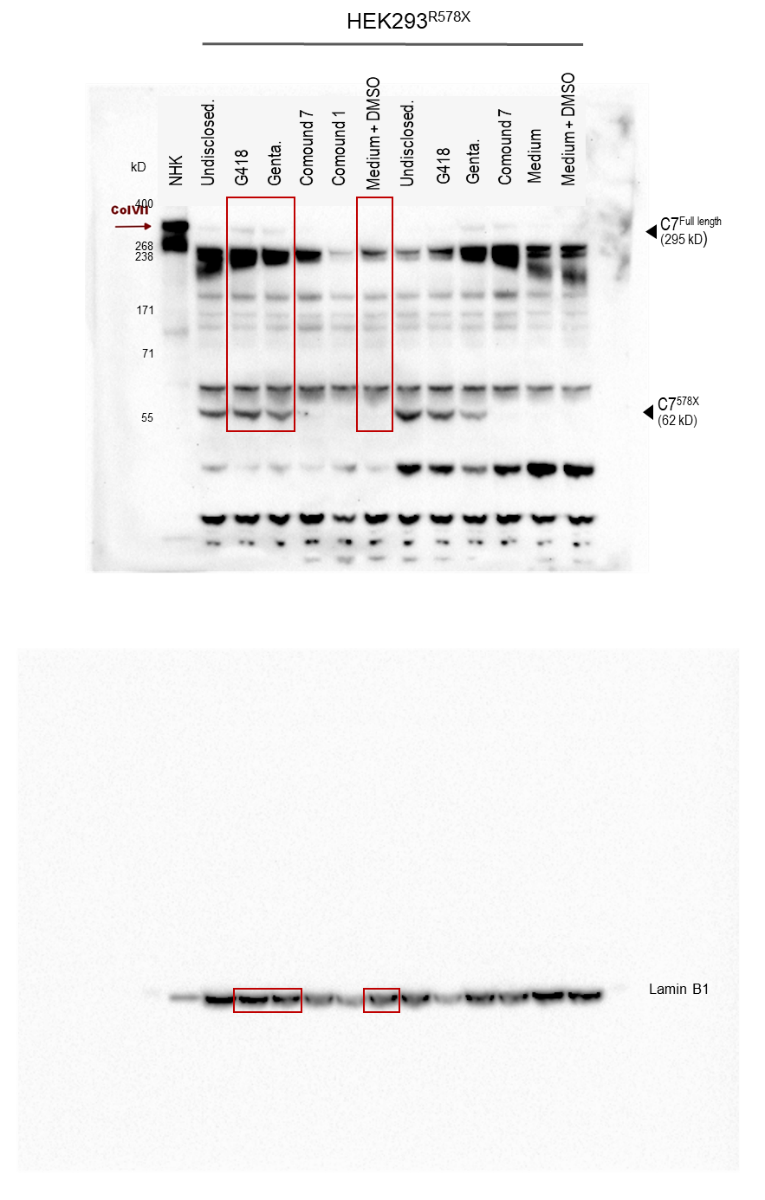
**

**Figure S2** Uncropped gels corresponding to western blots presented in **Fig 1c**. Areas shown in **Fig 1c** are indicated with red boxes (cropping was intended to highlight relevant bands in the figures shown in the main text).

**Table S1. Impact of representative hit compounds in the viability of NHK cells**

|  | **Cytotox.** |
| --- | --- |
|  | IC_50_ [μM] or  % inhibition of viability at [μM] |
| Genta | 1600 ± 280  (n=4) |
| G418 | 30.8% ± 3.4 at 40  (n=4) |
| **Comp. 1** | 19 ± 2.4  (n=4) |
| **Comp. 3** | 58% ± 13 at 50  (n=2) |
| **Comp. 7** | 19% ± 11 at 100  (n=4) |
| **Comp. 8** | 31 ± 4.1  (n=4) |

| Cytotox. = cytotoxicity measured as % inhibition of viability (ATP levels). |
| --- |

**
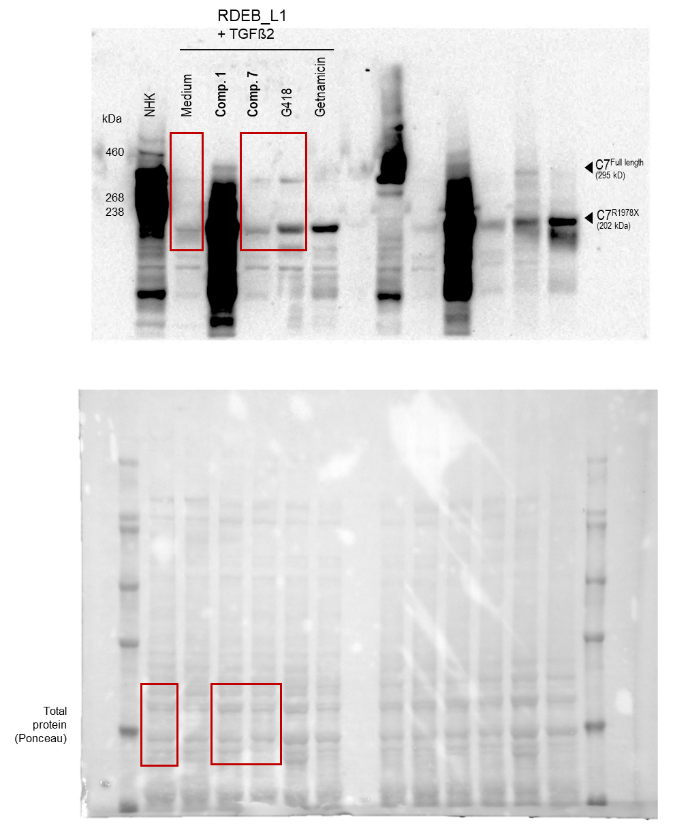

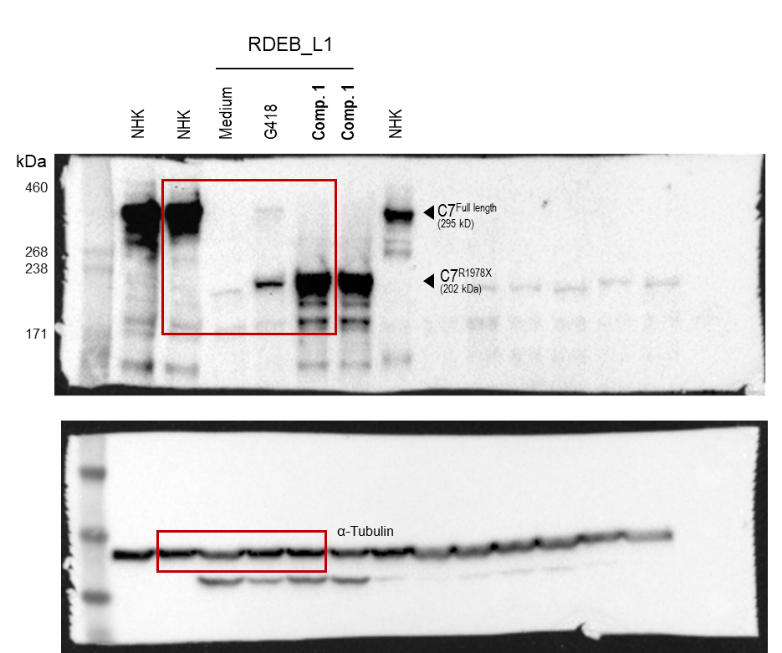
**

**
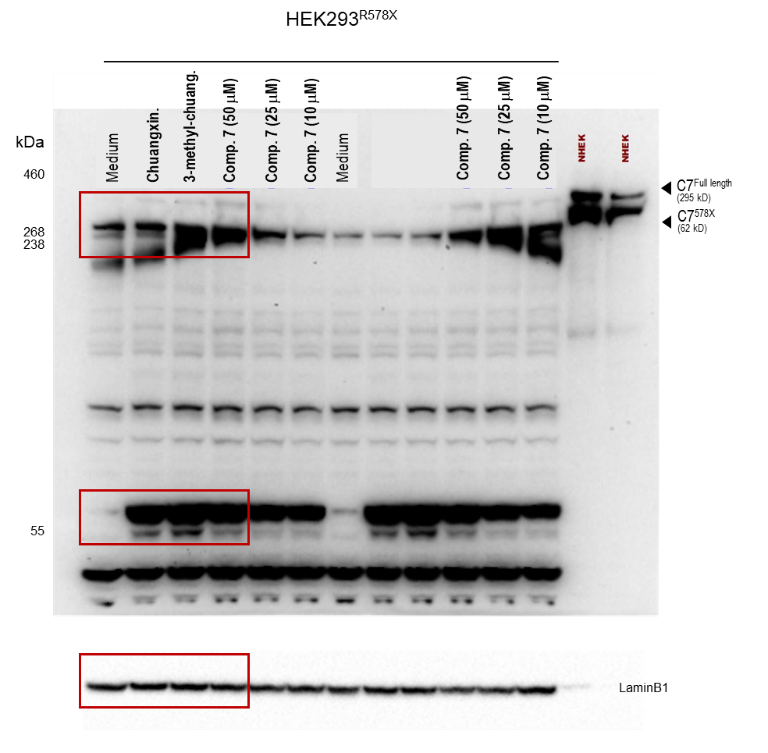
**

**Figure S3** Uncropped gels corresponding to western blots using duplicate samples presented in **Fig 2c**. Areas shown in **Fig 2c** are indicated with red boxes (cropping was intended to highlight relevant bands in the figures shown in the main text). An additional western blot not included in the main text is shown at the bottom left panel.

**
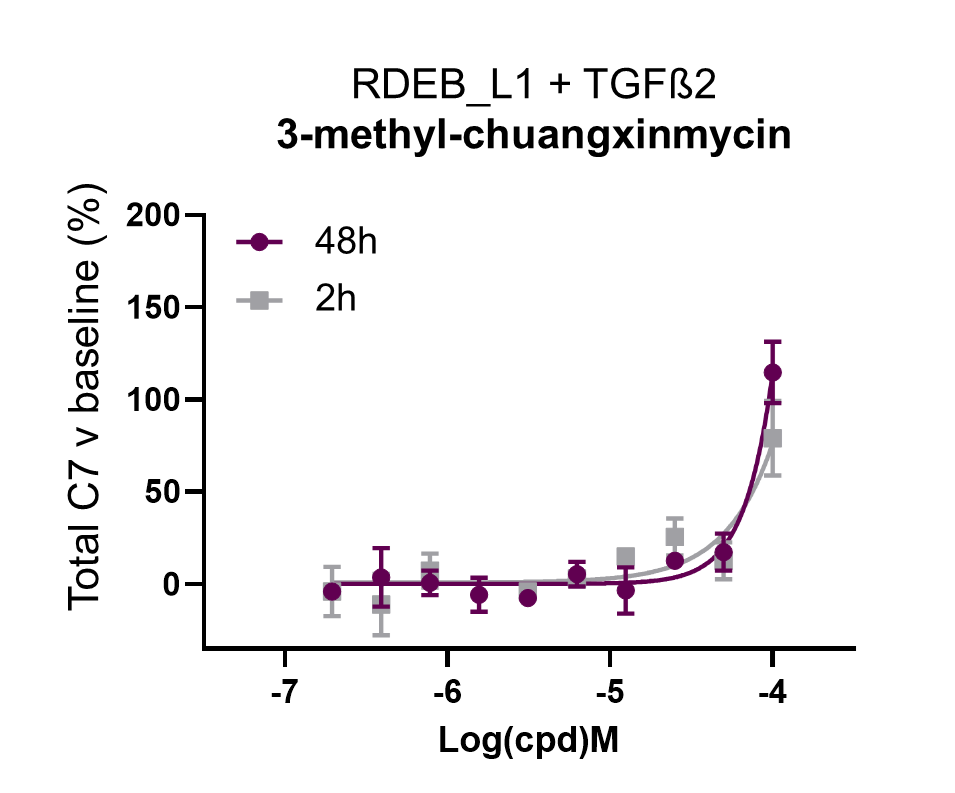
**

**Figure S4** Dose response AlphaLISA evaluation of **3-methyl-chuangxinmycin** in RDEB_L1 cells at 2 h and 48 h in the presence of 20 ng/mL of TGFß2. Representative result of n=3 experiments.


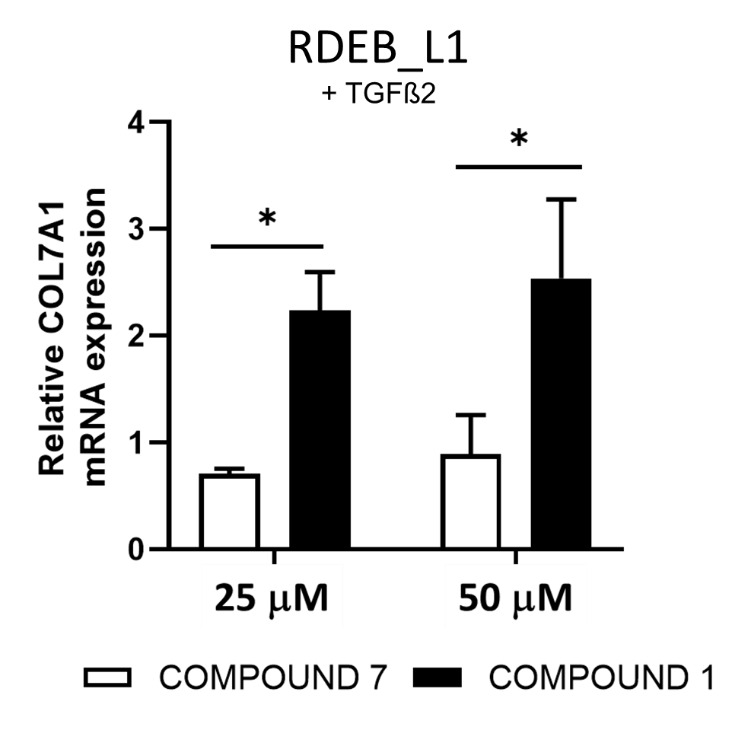


**Fig S5:** Comparative qPCR analysis of the induction of COL7A1 mRNA in RDEB_L1 cells by **compound** **7** and **compound** **1** in the presence of 20 ng/mL of TGFß2 (24 h), statistically significant differences are indicated by * p<0.05. Representative result of n=2.

**
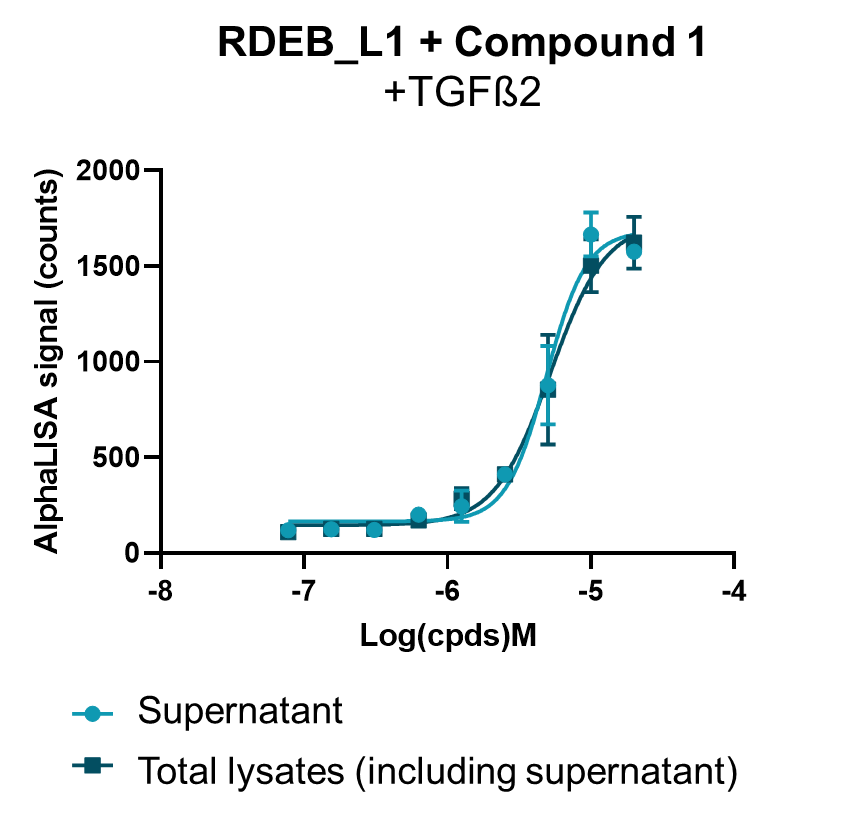
**

**Fig S6:** AlphaLISA dose response quantification of total C7 protein in RDEB_L1 cells treated with **compound 1** during 48 h in the presence of 20 ng/mL of TGFß2 in supernatant and total cell lysates including supernatant. 2 μL of supernatant were used for the supernatant dose response before lysis and dose response evaluation of the total lysates which included the remaining supernatant fraction.


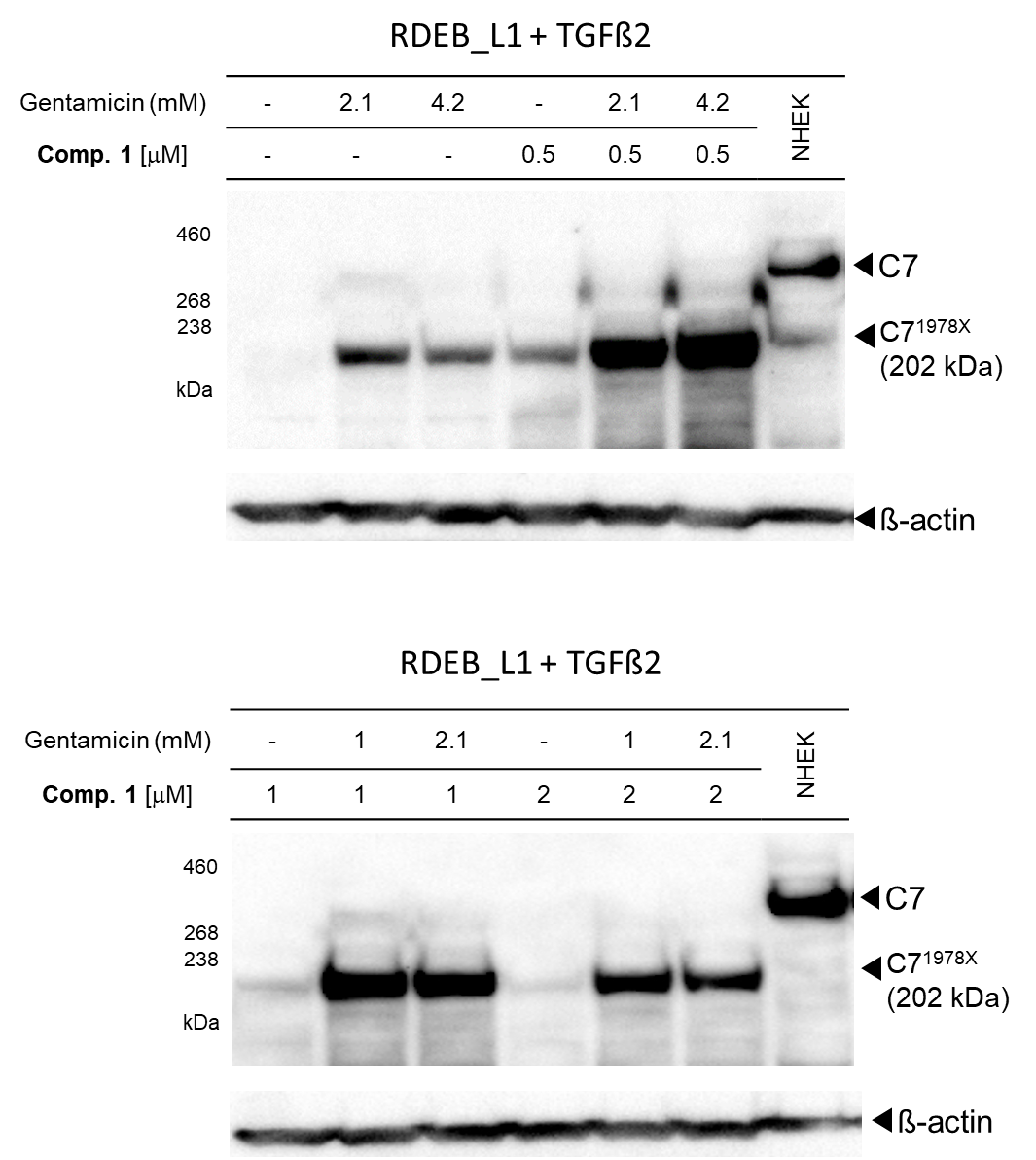


**Fig S7**: Western blot analysis of treated extracts with the indicated combinations of gentamicin and **compound 1** after incubation during 48 h in the presence of 20 ng/mL TGß2.

**
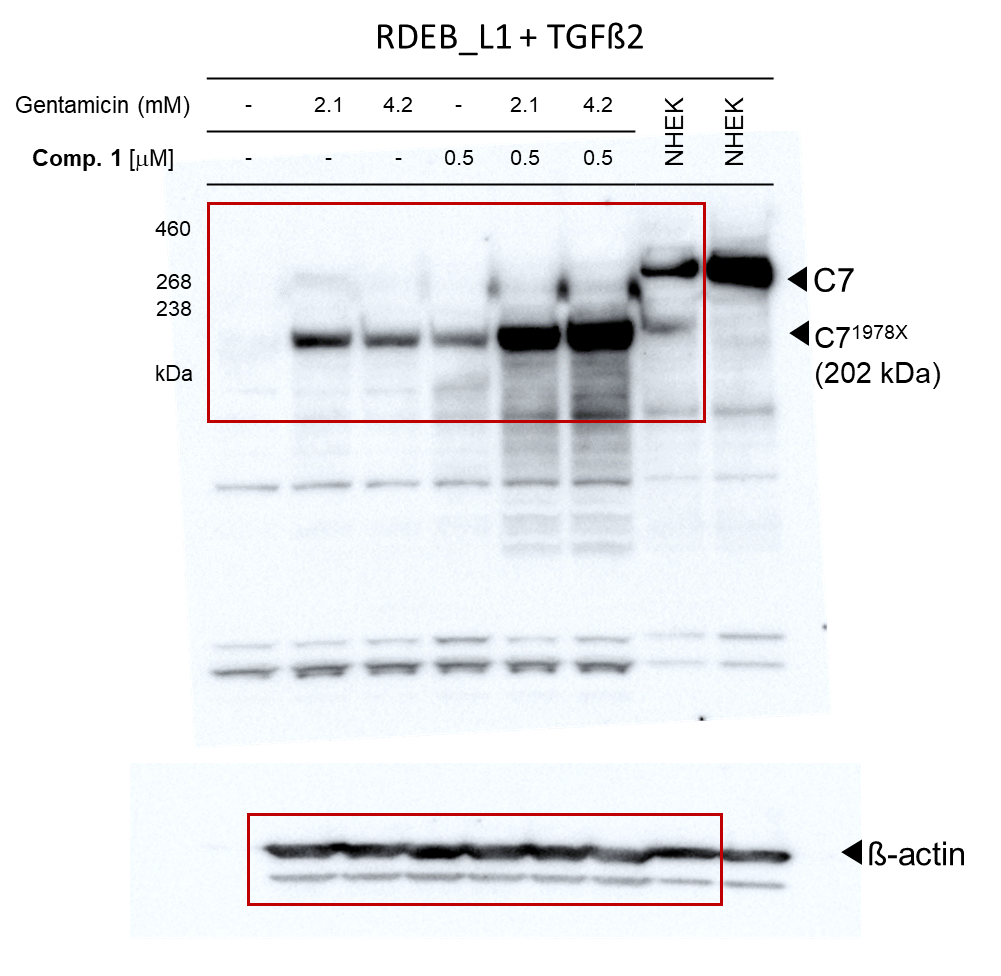
**

**
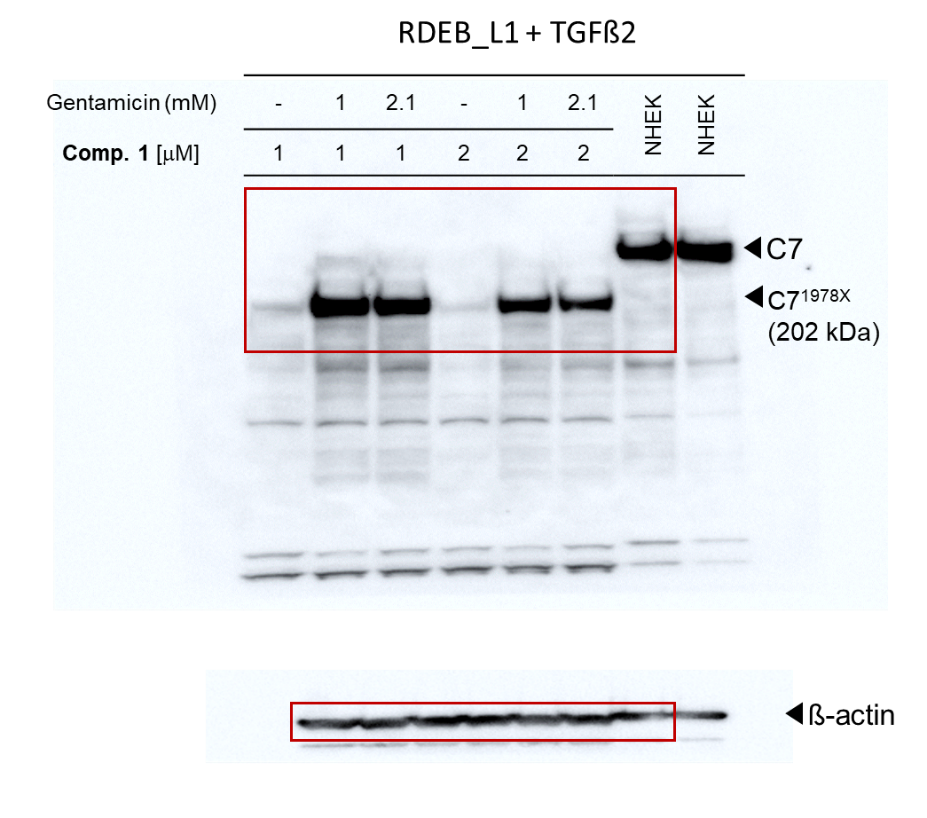
**

**Fig S8**: Uncropped gels corresponding to western blots presented in **Fig S7.** Areas shown in **Fig S7** are highlighted with red boxes (cropping was intended to highlight relevant bands in the figures shown in the main text).

**
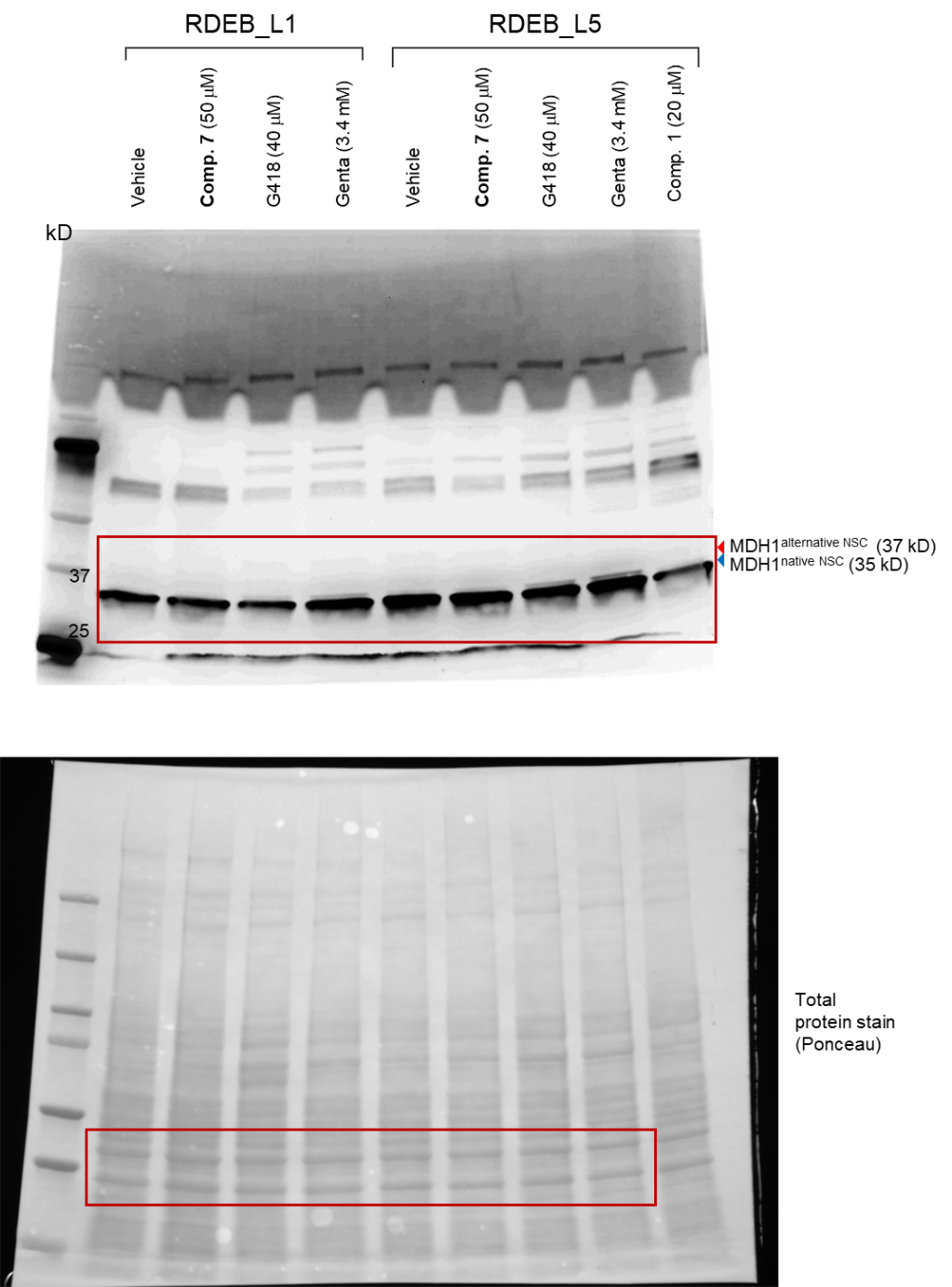
**

**Supplementary Fig S9.** Uncropped gels corresponding to western blots presented in **Fig 5.** Areas shown in **Fig 5** are highlighted with red boxes (cropping was intended to highlight relevant bands in the figures shown in the main text).

**Supplementary information Table S2**. Kinase selectivity panel evaluating **compound 7** selectivity at the indicated concentrations. Values show percentage of control activity at the indicated micromolar concentration of test compound. Highlighted in darker grey color, activities < 50%. Values shown are the mean of 2 replicate determinations.

**Supplementary information Table S3** General selectivity panel evaluating **compound 7** selectivity at the indicated concentrations. Values show percentage of inhibition of activity at the indicated concentrations. Highlighted in darker grey color, inhibitions > 50%. Values shown are the mean of 2 replicate determinations.
